# A deep learning framework for efficient pathology image analysis

**DOI:** 10.1038/s41467-026-74918-9

**Published:** 2026-07-01

**Authors:** Peter Neidlinger, Tim Lenz, Sebastian Foersch, Chiara M. L. Loeffler, Jan Clusmann, Marco Gustav, Lawrence A. Shaktah, Rupert Langer, Bastian Dislich, Lisa A. Boardman, Amy J. French, Ellen L. Goode, Andrea Gsur, Stefanie Brezina, Marc J. Gunter, Robert Steinfelder, Hans-Michael Behrens, Christoph Röcken, Tabitha Harrison, Ulrike Peters, Amanda I. Phipps, Giuseppe Curigliano, Nicola Fusco, Antonio Marra, Michael Hoffmeister, Hermann Brenner, Jakob Nikolas Kather

**Affiliations:** 1 Else Kroener Fresenius Center for Digital Health, Faculty of Medicine and University Hospital Carl Gustav Carus, TUD Dresden University of Technology, Dresden, Germany; 2 Institute of Pathology, University Medical Center Mainz, Mainz, Germany; 3 Department of Medicine I, Faculty of Medicine and University Hospital Carl Gustav Carus, TUD Dresden University of Technology, Dresden, Germany; 4 National Center for Tumor Diseases Dresden (NCT/UCC), Dresden, Germany; 5 Department of Medicine III, University Hospital RWTH Aachen, Aachen, Germany; 6 Institute of Pathology and Molecular Pathology, Kepler University Hospital, Johannes Kepler University Linz, Linz, Austria; 7 Institute of Tissue Medicine and Pathology, University of Bern, Bern, Switzerland; 8 Division of Gastroenterology and Hepatology, Mayo Clinic, Rochester, MN USA; 9 Division of Laboratory Genetics, Department of Laboratory Medicine and Pathology, Mayo Clinic, Rochester, MN USA; 10 Department of Quantitative Health Sciences, Division of Epidemiology, Mayo Clinic, Rochester, MN USA; 11 Center for Cancer Research, Medical University of Vienna, Vienna, Austria; 12 Nutrition and Metabolism Branch, International Agency for Research on Cancer, World Health Organization, Lyon, France; 13 Cancer Epidemiology and Prevention Research Unit, School of Public Health, Imperial College London, London, UK; 14 Division of Public Health Sciences, Fred Hutchinson Cancer Center, Seattle, WA USA; 15 Department of Pathology, University Hospital Schleswig-Holstein, Kiel, Germany; 16 Department of Epidemiology, University of Washington, Seattle, WA USA; 17 Division of New Drugs and Early Drug Development, European Institute of Oncology IRCCS, Milan, Italy; 18 Department of Oncology and Hemato-Oncology, University of Milan, Milan, Italy; 19 Division of Pathology, European Institute of Oncology IRCCS, Milan, Italy; 20 Division of Clinical Epidemiology and Aging Research, German Cancer Research Center (DKFZ), Heidelberg, Germany; 21 German Cancer Consortium (DKTK), German Cancer Research Center (DKFZ), Heidelberg, Germany; 22 Medical Oncology, National Center for Tumor Diseases (NCT), University Hospital Heidelberg, Heidelberg, Germany; 23 Pathology & Data Analytics, Leeds Institute of Medical Research at St James’s, University of Leeds, Leeds, UK

**Keywords:** Cancer imaging, Prognostic markers, Tumour biomarkers, Machine learning, Image processing

## Abstract

Artificial intelligence has transformed digital pathology by enabling biomarker prediction from high-resolution whole-slide images. However, current methods are computationally inefficient, processing thousands of redundant tiles per slide and requiring complex aggregation models. We introduce EAGLE (Efficient Approach for Guided Local Examination), a deep learning framework that emulates pathologists by selectively analyzing informative regions. EAGLE combines task-agnostic tile selection with detailed feature extraction and is benchmarked against leading slide- and tile-level foundation models across 43 tasks from nine cancer types spanning morphology, biomarker prediction, treatment response and prognosis. EAGLE outperforms patch aggregation methods by up to 23% and achieves the highest overall classification performance. It processes one slide in 2.27 s, reducing computational time by more than 99% compared with existing models. This efficiency supports rapid and auditable workflows by enabling review of the exact tiles used for each prediction and reducing dependence on high-performance computing. By reliably identifying informative regions and minimizing artifacts, EAGLE provides robust and auditable outputs, supported by systematic negative controls and attention concentration analyses. Its unified embedding enables rapid slide search, integration into multi-omics pipelines and emerging clinical foundation models.

## Abstract (web summary)

While AI methods have significantly advanced computational pathology, existing methods typically are very resource-intensive. Here, the authors develop EAGLE, a deep learning framework that emulates pathologists by selectively analyzing informative regions, and benchmark this model against leading slide- and tile-level foundation models across nine cancer types.

## Introduction

Artificial intelligence (AI) has significantly advanced computational pathology (CPath) by enabling the extraction of clinically relevant information from gigapixel-scale whole-slide images (WSIs)^1–6^. Existing methods use resource-intensive vision transformers trained with self-supervised learning (SSL) to encode detailed morphological features essential for diagnosis, prognosis, and treatment planning in oncology^7–11^. While these approaches have shown great promise across a wide range of tasks, their inefficiencies and limited scalability highlight the need for solutions that better align with real-world diagnostic workflows. Recently, pathology-specific multimodal large language models (MLLMs) have emerged as AI copilots for clinical decision-making, but they often underperform in biomarker prediction and the regulatory pathway for approving such models as medical devices remains uncertain^12–16^.

Current methods predominantly operate at the tile level, requiring the extraction and analysis of thousands of tiles per WSI, with datasets in this study averaging approximately 18,000 tiles per slide at a resolution of 0.5 µm/pixel (MPP). This computationally intensive process deviates from how pathologists evaluate slides, as they selectively focus on regions of interest^17–19^. Moreover, tile-wise features are aggregated into slide-level predictions using models trained separately for each task, limiting scalability and interpretability^8,20,21^. The complexity of these models often obscures the decision-making process, making it challenging to understand how predictions are derived and which tissue regions are influential. These systems also struggle in data-scarce scenarios, where tile selection often fails to identify the most relevant regions, leading to suboptimal predictions^22^. Such scenarios are often a clinical reality, for example during the evaluation of small biopsy specimens.

**Fig. 1:**
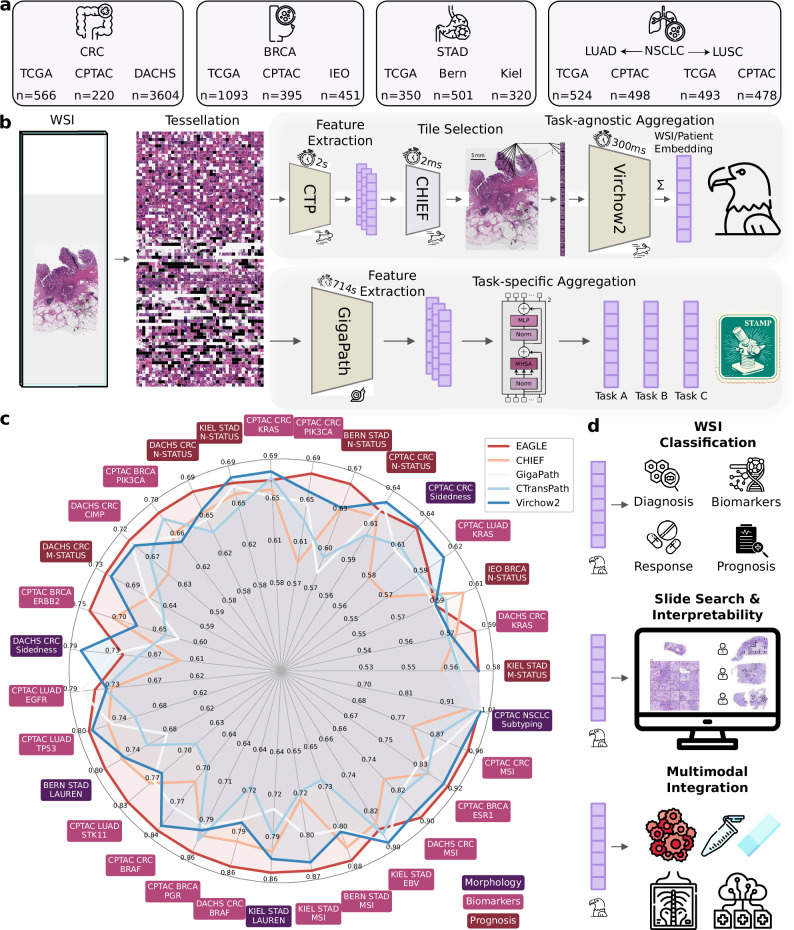
EAGLE framework. **a** Main benchmark overview: 9,528 whole-slide images (WSIs) from 13 cohorts spanning four cancer types. **b** Workflow comparison of EAGLE and conventional supervised pipelines. After tessellation, EAGLE applies CTransPath feature extraction, CHIEF tile selection, and Virchow2 encoding of 25 selected tiles to generate one averaged WSI embedding per patient. Supervised pipelines encode all tiles and aggregate them with task-specific models. Processing time per WSI is shown for EAGLE at 2 microns per pixel (MPP) and conventional supervised models at 0.5 MPP. **c** Mean AUROC across 31 computational pathology tasks for EAGLE, CHIEF, Prov-GigaPath, CTransPath, and Virchow2. Axes are normalized from 0.5 to the best AUROC for each task. **d** Example applications of WSI or patient embeddings, including classification, biomarker prediction, prognosis, retrieval, and multi-omics integration. Source data are provided as a Source Data file.

To address these limitations, we developed EAGLE (Efficient Approach for Guided Local Examination), a framework that emulates the diagnostic strategy of pathologists by focusing on a small, informative subset of tiles within WSIs. EAGLE combines CHIEF^23^, a pre-trained and task-agnostic model used for global tissue representation and guided tile selection, with Virchow2^24^, for detailed feature extraction from selected tiles. This combination substantially reduces computational demands while increasing performance (Fig. 1a–c). By selecting a small, reproducible subset of regions, EAGLE enhances auditability and scalability, particularly in biomarker prediction tasks where subtle morphological features are critical^25^. Unlike MLLMs, which emphasize multimodal interaction, EAGLE prioritizes efficient high-quality WSI analysis. Still, it can integrate with MLLMs to provide valuable inputs for enhanced contextual analysis. Through comprehensive evaluation against state-of-the-art models, including multiple instance learning (MIL) and slide-encoder approaches, we demonstrate the efficacy and robustness of EAGLE across 43 tasks spanning nine cancer types^8–10,23,24,26–30^.

## Results

### EAGLE improves upon state-of-the-art approaches

We first evaluated EAGLE within a previously established benchmarking framework encompassing 31 CPath tasks across breast (BRCA), colorectal (CRC), gastric (STAD), and non-small cell lung cancers (NSCLC)^22^. This dataset integrates diverse prediction objectives spanning morphology, biomarker and prognosis, and enables systematic comparison on large, truly external datasets. The benchmark includes both state-of-the-art slide-encoder and tile-encoder approaches: slide-level models (TITAN^30^, COBRA^29^, CHIEF^23^, Prism^26^, MADELEINE^27^, and Prov-GigaPath^9^) and tile encoders or foundation models (Virchow2^24^, CONCH v1.5^30^, CONCH^28^, Prov-GigaPath^9^, CTransPath^8^, Virchow^10^), which were aggregated using attention-based multiple instance learning (ABMIL)^21^, the standardized pipeline STAMP^18^, or simply by averaging all embeddings. All classifiers were trained using a five-fold cross-validation setup on The Cancer Genome Atlas (TCGA) data, resulting in five models per task. Each model was then evaluated on the full external test cohorts (CPTAC, Clinical Proteomic Tumor Analysis Consortium; DACHS, Darmkrebs: Chancen der Verhütung durch Screening; Kiel, University Medical Center Kiel; Bern, Inselspital Bern; IEO, European Institute of Oncology), ensuring external validation without data leakage (Supplementary Fig. 1c).

**Fig. 2:**
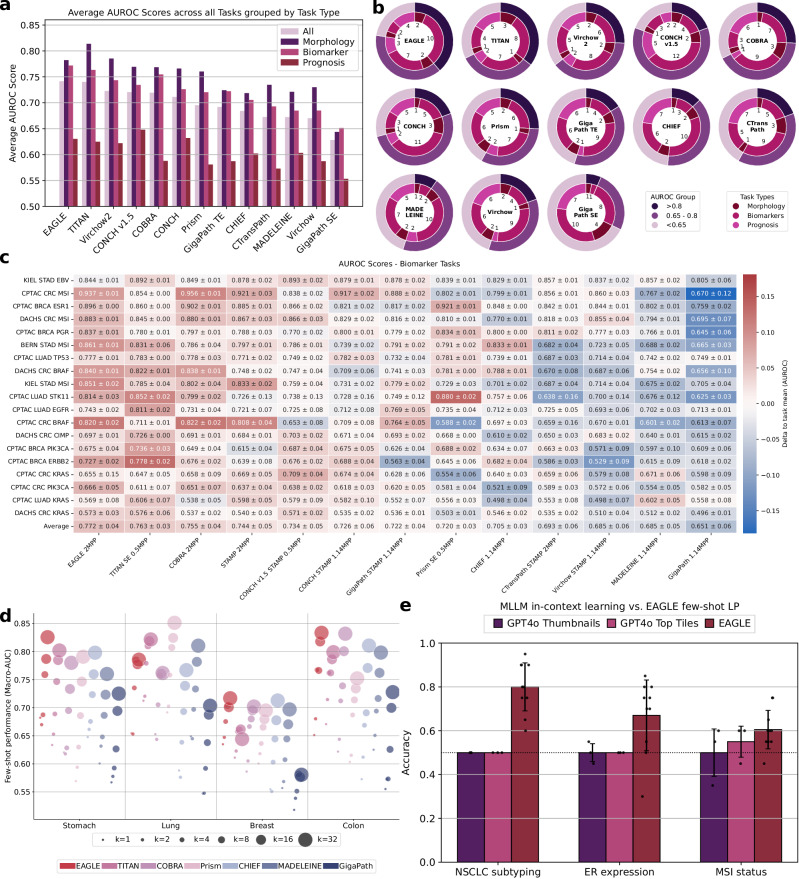
Comparative performance of EAGLE vs. tile- and slide-level foundation models. **a** Mean AUROC by task category across five folds for 13 models evaluated on 31 tasks. **b** Number of tasks per model with mean AUROC > 0.800, 0.650 to 0.800, or <0.650, grouped by task type. **c** Mean AUROC ± standard deviation (SD) across five folds for 19 biomarker tasks; colors show deviation from the task specific mean across models, with red indicating above mean and blue below mean performance. Tasks and models are ordered by mean AUROC. **d** Few-shot linear probing of slide encoders with logistic regression for *k* = 1, 2, 4, 8, 16, and 32 samples per class, using the top three binary tasks per cancer type; values show mean AUROC across *n* = 10 support-set resamplings. **e** EAGLE few-shot lin**e**ar probing versus GPT-4o in-context learning at *k* = 2 for BRCA ER expression, CRC MSI status, and NSCLC subtyping. Bars show mean accuracy, error bars SD, and dots individual runs. For EAGLE, *n* = 10 support-set resamplings were evaluated per task; for each GPT-4o input setting, *n* = 3 independent runs were performed per task. Source data are provided as a Source Data file.

Across all 31 tasks, EAGLE and TITAN achieved the highest average area under the receiver operating characteristic curve (AUROC) scores of 0.742 and 0.740, respectively. Following were Virchow2 in STAMP (0.723) and CONCH v1.5 in STAMP (0.721), suggesting that modern slide encoders often outperform strong tile-level baselines (Figs. 1c, 2a, Supplementary Fig. 2). EAGLE exceeded key AUROC thresholds more often than other models, surpassing 0.800 in 39% of tasks and 0.650 in 77% of tasks—higher than TITAN (35% and 68%) and Virchow2 (26% and 65%) (Fig. 2b). Looking at task-specific performance, EAGLE (0.772), TITAN (0.763), and COBRA (0.757) excelled on biomarker tasks, which predict molecular alterations or protein expression. In morphology tasks, which classify tumor location or histological patterns, TITAN achieved the highest performance (0.814), followed by Virchow2 (0.785) and EAGLE (0.782). Tile-level CONCH v1.5 in STAMP (0.648) held an advantage in prognosis, which assesses tumor spread, with EAGLE achieving the highest prognosis performance among slide-level frameworks (0.630) (Fig. 2c, Supplementary Fig. 3a, e). EAGLE also scored highest in three of the four cancer types—BRCA (0.737), CRC (0.710), and STAD (0.755)—while TITAN scored highest in lung (0.810) (Supplementary Fig. 3b, c, f). Beyond AUROC, EAGLE achieved the highest average area under the precision-recall curve (AUPRC) scores (0.566), followed by COBRA (0.556). In balanced accuracy, EAGLE and TITAN performed best with average scores of 0.657 and 0.655, respectively. TITAN held the highest average F1 scores (0.498), followed by EAGLE (0.490) (Supplementary Figs. 4–6).

We assessed the statistical significance of AUROC differences with two-sided DeLong’s tests, controlling the false discovery rate using the Benjamini–Hochberg procedure. For each patient, predictions from the five cross-validation models were averaged to yield a single ensemble score. Across all tasks and models, ensemble AUROC values were highly correlated with mean fold performance, with most model–task pairs lying close to the diagonal, indicating only marginal performance gains from ensembling (Supplementary Fig. 7a). Under these ensemble conditions, EAGLE achieved an average AUROC of 0.750, followed by TITAN (0.744) and CONCH (0.736) (Supplementary Fig. 8a). Statistical comparisons based on ensemble predictions confirmed that EAGLE significantly outperformed slide-level encoders across numerous tasks, with AUROC improvements exceeding 0.05 and occasionally 0.2 (Supplementary Fig. 7b). Similar trends were observed when comparing EAGLE with tile-level foundation models (Supplementary Fig. 7c). When restricting the comparison to the Virchow2 model aggregated through STAMP, ABMIL, or mean pooling, differences were smaller, with EAGLE outperforming in six task–model combinations and performing worse in four (Supplementary Fig. 7d). EAGLE’s strongest advantages were observed for DACHS CRC tasks (MSI, *BRAF*, *KRAS*, M-Status, and N-Status), potentially due to the large cohort size, which provided increased statistical power. In DACHS *BRAF* mutation prediction, EAGLE statistically outperformed all models except COBRA (Supplementary Fig. 8b).

Together, these analyses show that EAGLE achieves consistently high performance across tasks, cancer types, and external cohorts, often statistically outperforming state-of-the-art slide- and tile-level models. Its strong biomarker prediction accuracy and robust generalization across heterogeneous datasets highlight the quality and versatility of its representations.

### Design analysis of EAGLE and negative controls

**Fig. 3:**
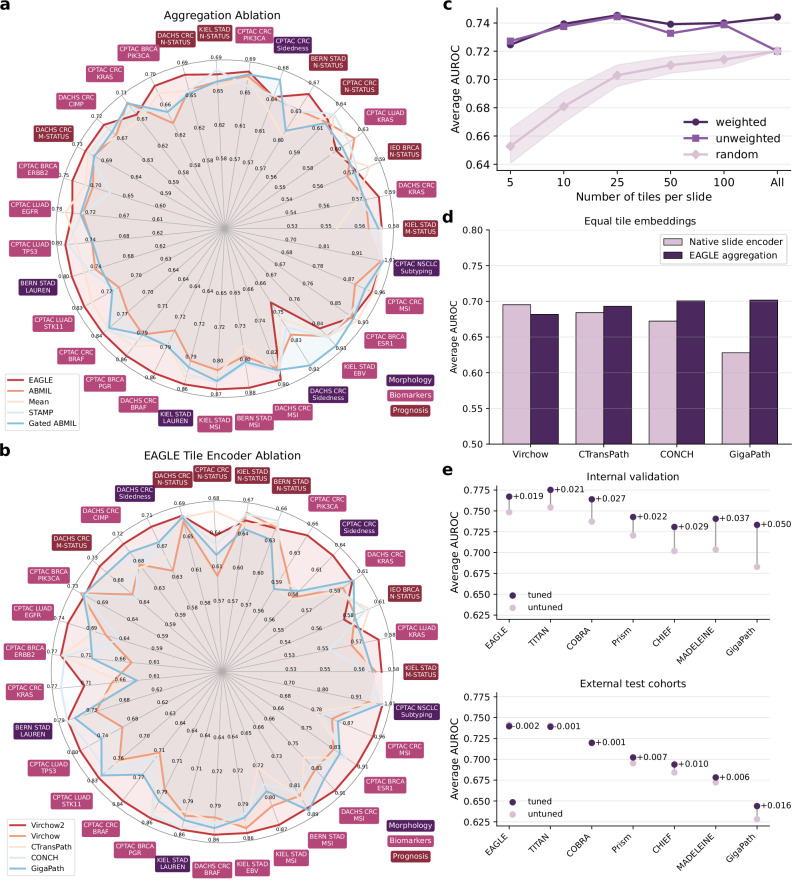
Ablation studies dissecting EAGLE design choices. **a** Alternative aggregation of Virchow2 tile embeddings at 2 microns per pixel (MPP) across 31 tasks: mean pooling, attention-based multiple instance learning (ABMIL), gated ABMIL, STAMP, and EAGLE. **b** Performance of five tile encoders within EAGLE across the same 31 tasks: Virchow2, Virchow, CTransPath, CONCH, and GigaPath. **c** Effect of tile number and selection strategy. CHIEF-based selection was evaluated for *N* = 5, 10, 25, 50, and 100 tiles per slide using unweighted or attention-weighted aggregation. Uniform random selection was repeated 100 times per *N*; lines show the mean and 95% interval. CHIEF-based selection outperformed all random replicates at every *N* (Monte Carlo p = 0.0099). **d** Mean AUROC across 31 tasks for native slide encoders and EAGLE built on the same tile embeddings. **e** Mean AUROC change after model tuning on 23 internal and 31 external validation tasks. Source data are provided as a Source Data file.

To understand which design choices yield EAGLE’s performance gains, we conducted extensive ablation studies. First, we compared five different options for aggregating Virchow2 tile embeddings at 2 MPP. EAGLE selects the 25 most informative tiles using CHIEF and averages their embeddings, whereas alternative approaches either aggregate tiles through supervised models (STAMP, ABMIL or gated ABMIL) or compute a simple mean across all tiles. Among these, EAGLE achieved the highest mean AUROC (0.742), followed by gated ABMIL (0.723), STAMP (0.723), mean pooling (0.720), and regular ABMIL (0.711), confirming that EAGLE provides the most effective tile aggregation strategy. (Fig. 3a, Supplementary Fig. 9a). Second, we varied the tile encoder while keeping EAGLE aggregation fixed. Performance remained robust across encoders, indicating that EAGLE benefits from stronger tile features but does not rely on a single backbone. We then asked whether EAGLE can replace bespoke slide encoders trained for specific tile embeddings. Across four backbones, EAGLE exceeded the native slide encoders for three and trailed slightly for one. Gains were largest with GigaPath (+12%) and CONCH (+4%), modest with CTransPath (+1%), and negative with Virchow (−2%). Pooled across all comparisons, EAGLE led in about two thirds of tasks with a small increase in mean AUROC, supporting its role as an effective universal slide-level aggregator (Fig. 3b, d, Supplementary Fig. 9b, c). Third, we systematically analyzed how the number and selection strategy of tiles affect EAGLE’s performance. We compared three approaches: CHIEF-based selection of the top-ranked tiles aggregated either (i) by simple averaging (unweighted) or (ii) by CHIEF-derived attention weights (weighted), and (iii) random tile selection as a negative control repeated 100 times per tile budget. For both CHIEF-based strategies, performance increased up to 25 tiles, where the highest AUROC was reached (0.745 for unweighted, 0.744 for weighted), indicating that a small, targeted subset captures most predictive information. Notably, using only the top five selected tiles (0.727) already outperformed mean pooling of all embeddings (0.720). A 100-replicate random baseline defined an empirical null distribution for each tile budget (*N* = 5, 10, 25, 50, 100), enabling uncertainty estimation and Monte Carlo testing. The mean AUROC of the replicate distribution increased with larger tile budgets, approaching the mean pooling baseline as *N* increased. For every *N*, CHIEF-based selection exceeded the maximum random replicate, corresponding to a Monte Carlo *p*-value of 1/101 and excluding the hypothesis that any reasonable unguided subset performs comparably (Fig. 3c, Supplementary Table 1). Because weighted and unweighted aggregation performed similarly at 25 tiles, we adopted equal averaging of the top 25 tiles as the default to enable transparent region-level auditability (Fig. 3c, Supplementary Fig. 9e). To address intra-patient heterogeneity, we next compared two methods for creating a patient-level representation. Early fusion aggregates across all slides jointly before forming a single patient embedding. Late fusion averages slide-level embeddings after separate aggregation per slide. On average the difference was small (mean Δ = −0.005), although early fusion tended to help for morphology and prognosis tasks while biomarker tasks showed little change. (Supplementary Fig. 9d). Moreover, we examined how magnification choices influence performance. While EAGLE and COBRA each peaked at 2 MPP (0.742 and 0.719, respectively), TITAN and Prism favored 0.5 MPP (0.740 and 0.695). For Prov-GigaPath (0.628), CHIEF (0.684) and MADELEINE (0.672), 1.14 MPP worked best, implying that the optimal resolution differs between models (Supplementary Fig. 9f). Finally, to address concerns about potential bias from suboptimal hyperparameters, we performed extensive MLP hyperparameter tuning using only internal validation cohorts. On internal data, tuning led to modest AUROC gains of 0.02–0.03 on average, with larger improvements for lower-performing models such as GigaPath (+0.05) and MADELEINE (+0.037). However, these gains did not generalize to external test cohorts, where performance changes were negligible (<0.01 on average across models) (Fig. 3e). Correlation analysis between internal and external AUROC deltas (*R²* = 0.04) confirmed that tuning improvements largely reflect overfitting rather than transferable optimization. These findings indicate that extensive hyperparameter tuning provides little benefit for large embedding-based models in CPath (Supplementary Fig. 10).

Together, these investigations highlight that EAGLE’s performance gains arise from its architecture and aggregation strategy rather than hyperparameter bias. Selecting and averaging the most informative tiles provides clear advantages over established aggregation methods, slide encoders, and random or exhaustive tile inclusion. EAGLE’s approach remains robust across magnifications and effectively integrates multiple slides per patient, underscoring the stability and generalizability of its representations.

### End-to-end weak supervision drives attention toward near-uniform aggregation

**Fig. 4:**
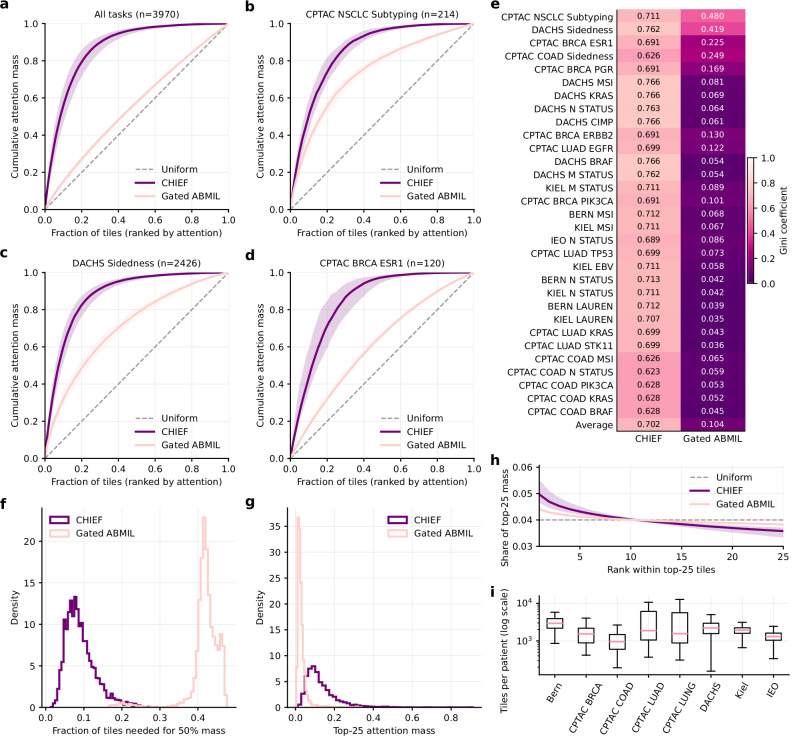
Quantitative attention concentration analyses. **a**–**d** Lorenz curves of cumulative attention mass as a function of the fraction of tiles ranked by attention. **a** is aggregated across all tasks, with each matched patient counted once by averaging task-specific rank profiles rather than tile identities; **b**–**d** show representative task-specific curves for CPTAC NSCLC subtyping, DACHS sidedness, and ER expression in CPTAC BRCA. Solid lines indicate median patient-level curves and shaded bands indicate interquartile ranges (IQRs). The uniform reference is y = x. **e** Gini coefficient heatmap by task and model, including an average row. **f** Distribution of the fraction of tiles required to accumulate 50% of total attention mass, computed per patient and shown as density curves. **g** Distribution of cumulative attention mass within the top 25 tiles, shown per patient. **h** Contribution profile of the top 25 ranks, showing the share of top-25 mass contributed by each rank position. Solid lines indicate median patient-level shares and shaded bands indicate IQRs across matched patients (*n* = 3970). The uniform reference is 1/25. **i** Tile count distributions for external test cohorts on a log scale, illustrating what 25 selected tiles represent as a fraction of available tissue area. Boxes span the 25th to 75th percentiles, center lines indicate medians, and whiskers extend to the most extreme values within 1.5× IQR. Cohort-specific patient numbers are reported in Supplementary Table 5. Source data are provided as a Source Data file.

To directly test whether EAGLE’s fixed, task-agnostic tile selection is a principled response to a measurable limitation of end-to-end weak supervision, we quantified attention concentration for CHIEF, ABMIL, and a strengthened gated ABMIL baseline. CHIEF attention scores were obtained from the frozen CHIEF model operating on CTransPath embeddings, whereas ABMIL and gated ABMIL attention scores were obtained from task-specific models trained on Virchow2 embeddings. For each method, we computed Lorenz curves of cumulative attention mass as a function of the fraction of tiles ranked by attention, along with summary statistics including the Gini coefficient, the fraction of tiles required to accumulate 50% and 80% of the attention mass, and top-k mass metrics (Fig. 4, Supplementary Table 2, Supplementary Fig. 11). These analyses were performed per patient and then aggregated across tasks and folds by averaging attention rank profiles rather than tile identities, preventing trivial averaging effects.

Across tasks, CHIEF induced a stable non-uniform saliency ordering. The median fraction of tiles required to accumulate 50% of attention mass was 8.4% for CHIEF, compared with 44.1% for ABMIL and 42.0% for gated ABMIL, with corresponding 80% mass fractions of 20.5%, 75.8%, and 73.6% (Fig. 4a, f, Supplementary Table 2). This pattern was strongest for subtle biomarker endpoints, where (gated) ABMIL attention frequently remained broadly distributed and approached mean pooling behavior, but it became more selective for overt morphology endpoints such as NSCLC subtyping and CRC sidedness (Fig. 4b–d). Consistent with these concentration curves, the mean Gini coefficient was 0.702 for CHIEF versus 0.087 for ABMIL and 0.104 for gated ABMIL (Fig. 4e).

**Fig. 5:**
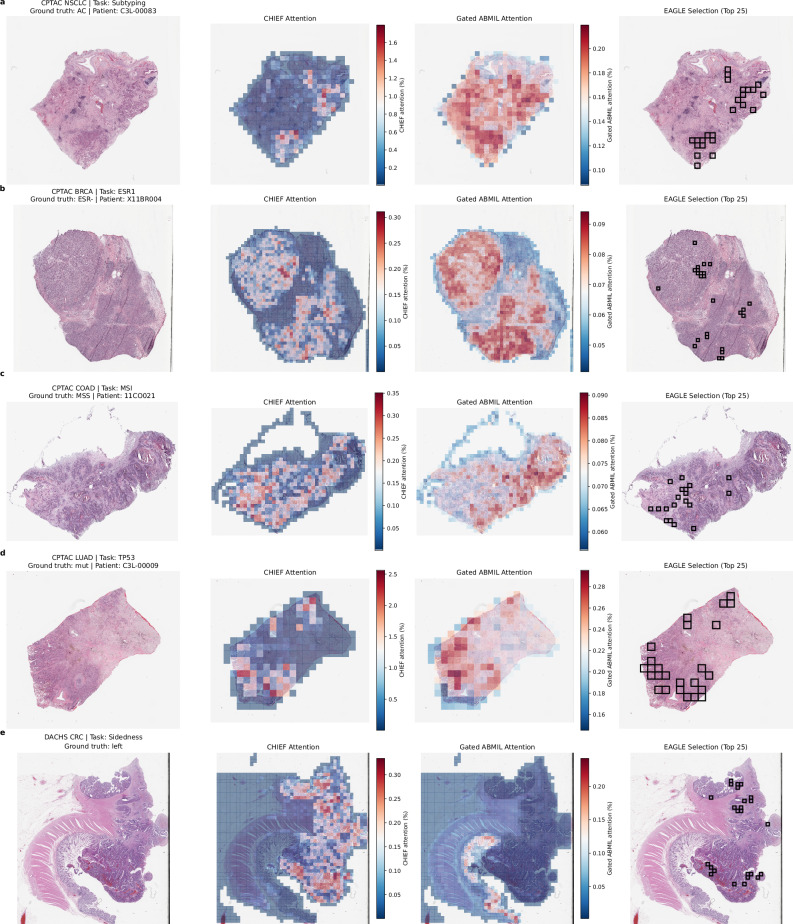
Direct visualization of attention maps and EAGLE top-25 overlays. For each representative whole-slide image, columns show a low-resolution hematoxylin and eosin (H&E) thumbnail, the CHIEF attention heatmap computed from frozen CHIEF on CTransPath embeddings, the gated attention-based multiple instance learning (gated ABMIL) heatmap from a task-specific model trained on Virchow2 embeddings, and the EAGLE tile set, shown as black boxes marking the exact 25 tiles selected by CHIEF and used to construct the EAGLE representation. Panels show representative external test cases for non-small cell lung cancer subtyping (**a**), ER expression in CPTAC BRCA (**b**), MSI status in CPTAC COAD (**c**), TP53 mutation in CPTAC LUAD (**d**), and colorectal cancer sidedness in DACHS (**e**). Heatmaps were softmax-normalized within each slide, and color scales were normalized per model and panel. Matched-scale controls are shown in Supplementary Fig. 12, and standard ABMIL examples in Supplementary Fig. 13.

To assess whether CHIEF-based selection is dominated by very few tiles, we quantified cumulative attention mass in the top 1, 2, and 25 ranked tiles. These metrics indicate that CHIEF attention is concentrated yet not driven by a single region (Fig. 4g, h, Supplementary Table 2). Finally, we provide direct visual comparisons on the same slides, showing CHIEF and gated ABMIL attention maps with explicit overlays of the exact top 25 tiles used by EAGLE, which makes the difference between concentrated saliency rankings and diffuse task-specific attention immediately observable (Fig. 5, Supplementary Figs. 12–13).

### Generalization to additional cancer types and complex clinical tasks

**Fig. 6:**
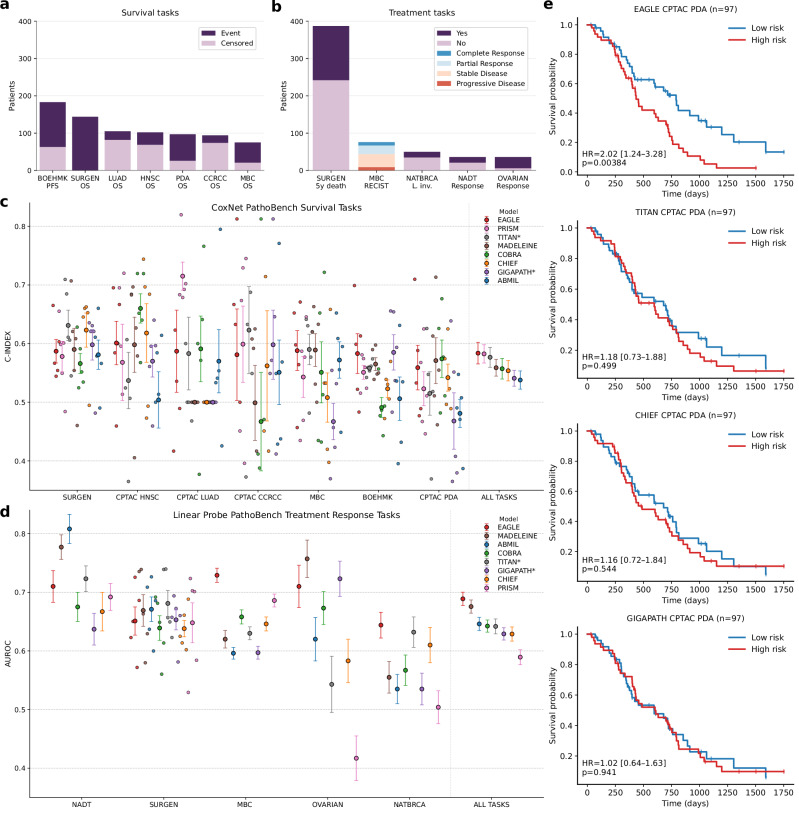
Extended evaluation of EAGLE on PathoBench tasks. **a** Overview of seven survival prediction tasks in the PathoBench extension, including six overall survival (OS) and one progression-free survival (PFS) endpoint. Numbers of events and censored cases are shown for each task. **b** Overview of five treatment response tasks, showing the number of patients per response category, including 5-year mortality, response evaluation criteria in solid tumors (RECIST) classes, lymphovascular invasion, and binary treatment response. **c** Concordance index (C-index) for seven survival tasks using the CoxNet implementation in PathoBench, comparing eight models. Dots indicate fold-level test-set values, and larger symbols with error bars indicate mean ± standard error of the mean (s.e.m.) across *n* = 5 cross-validation folds. The unit of analysis was the individual patient case. The aggregate column denotes the unweighted mean across tasks. TITAN and GigaPath require spatial coordinates and were therefore evaluated by averaging slide-level embeddings to obtain patient-level representations; all other models used early fusion before pooling. **d** AUROC for five treatment response tasks. For SURGEN 5-year mortality, dots indicate fold-level test-set values and larger symbols show mean ± s.e.m. across *n* = 5 cross-validation folds. For MBC RECIST, POST-NAT-BRCA lymphovascular invasion, NADT response, and OVARIAN response, larger symbols show mean ± s.e.m. across *n* = 50 Monte Carlo cross-validation folds; individual folds are omitted for clarity. The unit of analysis was the individual patient case for SURGEN, MBC, NADT, and OVARIAN, and the individual slide for POST-NAT-BRCA. **e** Kaplan-Meier analysis for EAGLE, TITAN, CHIEF, and GigaPath on CPTAC pancreatic ductal adenocarcinoma. Patients were dichotomized at the median predicted risk score; hazard ratios were estimated with Cox models and *P* values with two-sided log-rank tests. Source data are provided as a Source Data file.

To evaluate EAGLE’s generalization beyond the benchmarking tasks, we next applied it to the PathoBench dataset, which includes 12 survival and treatment response prediction tasks covering five additional cancer types^31,32^. In contrast to the benchmarking framework, PathoBench does not include external cohorts, and training and testing were performed within the same cohort, often with smaller sample sizes (Fig. 6a, b). The seven survival tasks comprised one progression-free survival endpoint and six overall survival endpoints, whereas the five treatment-response tasks assessed binary or multi-class outcomes including 5-year mortality, response evaluation criteria in solid tumors (RECIST)-based therapy response, lymphovascular invasion, and drug sensitivity.

Across the seven survival prediction tasks, EAGLE achieved the highest mean concordance index (C-index = 0.584), followed by Prism (0.582) and TITAN (0.577) (Fig. 6c). For treatment-response prediction, EAGLE again outperformed all other approaches, reaching a mean AUROC of 0.689, while MADELEINE (0.676) and ABMIL (0.646) were the second- and third-best performing models, respectively (Fig. 6d). To further assess prognostic stratification, we analyzed model-derived risk scores obtained from the CoxNet survival models. For each model, patients were divided at the median predicted risk score into high- and low-risk groups, and Kaplan–Meier curves were generated to visualize survival separation. On the CPTAC PDA cohort, EAGLE achieved clear stratification between high- and low-risk patients (hazard ratio (HR) = 2.02, 95% CI [1.24, 3.28], *p* = 0.0038), whereas TITAN (HR = 1.18, 95% CI [0.73, 1.88]), CHIEF (HR = 1.16, 95% CI [0.72, 1.84]), and GigaPath (HR = 1.02, 95% CI [0.64, 1.63]) showed no significant separation (*p* > 0.5) (Fig. 6e). Across additional survival tasks, EAGLE frequently showed stronger risk group separation than other models, including high hazard ratios on CPTAC CCRCC (HR = 2.30, 95% CI [0.94, 5.64]) and HNSC (HR = 1.88, 95% CI [0.91, 3.89]) (Supplementary Fig. 14).

Overall, these results demonstrate that EAGLE generalizes effectively to new cancer types and clinically relevant endpoints, including complex survival and treatment response tasks.

### Efficiency and performance in data-scarce scenarios

**Fig. 7:**
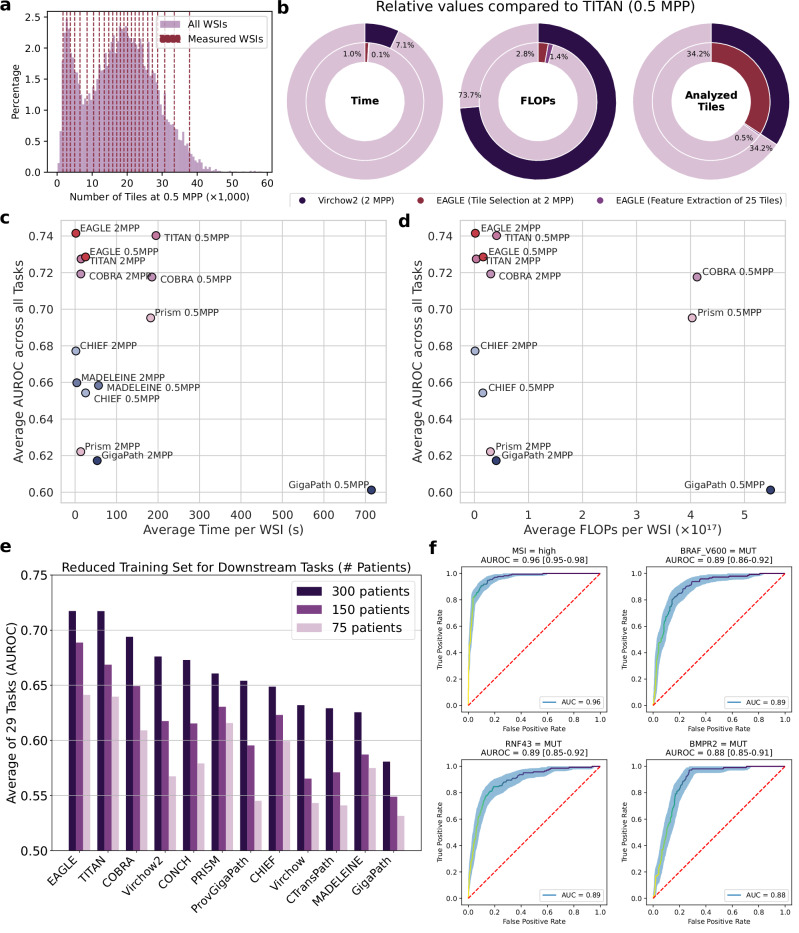
Computational efficiency and performance in scarce-data settings. **a** Tile distribution across 9,528 whole-slide images (WSIs) at 0.5 microns per pixel (MPP); 25 WSIs sampled from the 2nd to the 98th percentile were used for timing experiments. **b** Runtime, floating point operations (FLOPs), and number of analyzed tiles for processing these 25 WSIs with EAGLE and Virchow2 at 2 MPP, normalized to TITAN at 0.5 MPP. EAGLE is separated into tile selection by CTransPath and CHIEF and feature extraction of the 25 selected tiles by Virchow2. Runtime (**c**) and FLOPs (**d**) versus mean AUROC across 31 tasks. **e** Mean AUROC across 29 tasks for seven slide encoders and their tile-based counterparts trained with 300, 150, or 75 patients. **f** ROC curves for MSI, *BRAF V600*, *RNF43*, and *BMPR2*, with 95% bootstrap confidence intervals from 1000 resamples. Source data are provided as a Source Data file.

We next quantified computational requirements for the major pipeline steps, measuring average inference times for tile extraction and slide encoding using 25 representative slides of the benchmarking dataset (Fig. 7a). All measurements were performed on a workstation equipped with an L40 GPU (48GB), considering only model inference times. The slowest step is the tile-level feature extraction. On average, CTransPath at 0.5 MPP required 25.4 s/WSI and only 2.01 s/WSI at 2 MPP. By contrast, CONCH v1.5 at 0.5 MPP cost 191.85 s/WSI, and Prov-GigaPath 16 min/WSI (Supplementary Table 3). The faster slide encoding took on average 0.36 ms for CHIEF (2 MPP), while TITAN (0.5 MPP) took 3296 ms and Prism (0.5 MPP) 153 ms. EAGLE first processed each WSI with CTransPath at 2 MPP (2.01 s/WSI), applied CHIEF (0.36 ms/WSI), and selected 25 key tiles. Those tiles were then re-extracted with Virchow2 (0.26 s/WSI). On average, ~2% of tiles are reprocessed in detail at 2 MPP using Virchow2 (or ~0.1% at 0.5 MPP). When plotted against overall AUROC, EAGLE obtained the highest performance while ranking among the most efficient in terms of run time and floating point operations (FLOPs) (Fig. 7b–d). Prov-GigaPath, in contrast, was both the most time-consuming and the poorest-performing. TITAN, though competitive with EAGLE, required markedly more compute (second slowest overall).

Medical imaging datasets often contain limited samples per class, prompting few-shot or low-resource scenarios. We first assessed linear probing on EAGLE (and other slide encoder) embeddings with *k* = 1, 2, 4, 8, 16, 32 samples per class using logistic regression. To ensure meaningful comparisons, we selected only the top three binary tasks per cancer type based on AUROC in the main results, as linear probing performs poorly on low-performance tasks, increasing randomness and diluting differences between models. While performance dips in this challenging setting, both EAGLE and TITAN consistently outperformed others. EAGLE performed best in all cancer types except lung, where Prism excelled. With *k* = 1, 2, 4, TITAN led across all 12 tasks, followed closely by EAGLE, with both models achieving notable gaps over others (e.g., *k* = 4: TITAN/EAGLE AUROC = 0.72, Prism = 0.68, Prov-GigaPath = 0.58). At *k* = 8, 16, 32, EAGLE surpassed all other models, showcasing the advantage of its focused tile selection in low-data settings (Fig. 2d, Supplementary Fig. 15b, d).

We further evaluated EAGLE and other slide-encoder vs. tile-encoder models by training regular multilayer perceptron (MLP) classifiers on 300, 150, or 75 patients. Over 29 tasks with sufficient data, EAGLE maintained the highest average AUROC across all three subsets, particularly at 150 patients (0.689 vs. 0.669 for TITAN and 0.618 for the best tile encoder) (Fig. 7e, Supplementary Fig. 15a, c). This robust performance gap highlights how slide-level embeddings support model training when only small cohorts are available. To demonstrate EAGLE’s practicality for rare-biomarker discovery, we examined almost 300 biomarkers in a multicenter cohort of over 1000 patients. The entire processing time from the raw images to the final predictions took <24 h and we detected 20 biomarkers with AUROCs >0.800 (Fig. 7f, Supplementary Table 4).

Together, these efficiency metrics confirm that EAGLE’s guided, two-step approach minimizes computation without compromising performance and scales well to large or multi-task pathology pipelines. EAGLE’s adaptability in data-constrained contexts indicates that the combination of slide-level embeddings, selective tile processing, and efficient feature extraction yields robust performance when training data are limited.

### Versatility and interpretability of EAGLE

**Fig. 8:**
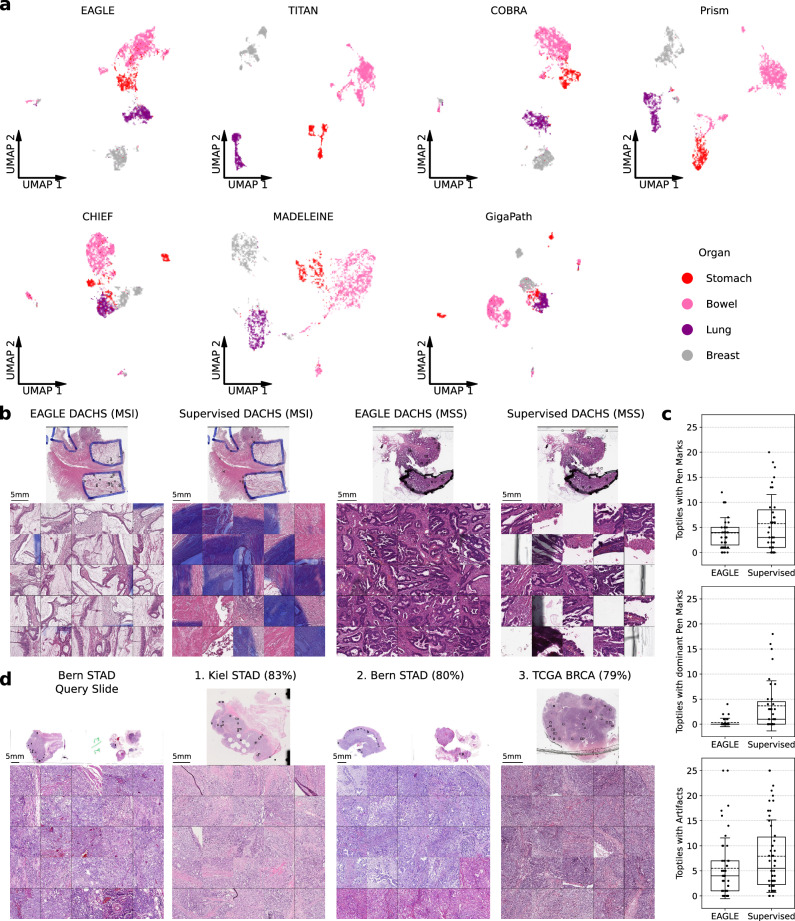
Versatility and interpretability. **a** Uniform Manifold Approximation and Projection (UMAP) of slide embeddings from EAGLE and the tested slide encoders, with four tissue types color-coded. **b** Top 25 tiles selected by EAGLE and by the supervised baseline for microsatellite instability (MSI) prediction in two representative DACHS slides (one MSI, one microsatellite stable (MSS)); the supervised baseline frequently selects artifacts, whereas EAGLE preferentially selects tissue-relevant regions. **c** Artifact prevalence among top tiles from 50 randomly selected DACHS whole-slide images (WSIs). Pen-mark and dominant pen-mark analyses included the 35 WSIs containing pen marks; other-artifact analysis included all 50 WSIs. Boxes span the interquartile range, center lines denote medians, points denote individual WSIs, dashed lines denote means, and error bars denote standard deviations. **d** Example slide retrievals in the EAGLE embedding space; percentages indicate cosine similarity between the Bern query embedding and its three nearest neighbors. Source data are provided as a Source Data file.

EAGLE generates a single, compact embedding per whole-slide or patient by restricting computation to a small number of highly informative tiles. The selection of only 25 top tiles, those identified as most relevant by the model, makes it explicit which tissue regions are used to construct the slide representation and can therefore be reviewed. To illustrate this interpretability advantage, we examined a subset of 50 randomly selected WSIs from the DACHS CRC cohort for MSI prediction, where 35 WSIs contained pen marks. Across all top tiles of these 35 WSIs, pen marks were present in 16% of EAGLE-selected tiles compared to 23% of tiles selected by the supervised baseline, which uses Virchow2 tile embeddings aggregated with STAMP. Notably, when considering only dominant pen marks (covering >50% of the tile), EAGLE almost completely avoided them (1%), whereas the supervised baseline selected those tiles in 15% of cases, even though they did not provide valuable information. Expanding the analysis to include all artifacts (e.g., tissue folds, slide edges, air bubbles, oil drops, scratches, pen marks, foreign objects, dark spots, and out-of-focus regions), EAGLE-selected tiles showed artifacts in 22% of cases, compared to 32% in the tiles selected by the supervised baseline (Fig. 8b, c). Furthermore, a board-certified pathologist inspected top selected tiles and found that EAGLE reliably focused on the most representative tumor tissue of each case. In contrast, the supervised baseline often focused on a mixture of healthy, tumorous and artifact-rich tiles, providing puzzling and sometimes contradictory features for prediction (Supplementary Fig. 16a). This capacity to confirm which tiles are chosen could further support clinical acceptance.

We then visualized slide embeddings with Uniform Manifold Approximation and Projection (UMAP)^33^. We noted that TITAN formed well-separated clusters by cancer type, while EAGLE displayed moderate clustering and surpassed CHIEF and Prov-GigaPath Slide Encoder in capturing morphological diversity (Fig. 8a). To further evaluate embedding structure, we compared UMAP projections of CHIEF and EAGLE across 29 TCGA cohorts, observing clearer separation with EAGLE (Supplementary Fig. 16b). Moreover, we explored slide retrieval applications, wherein a query WSI’s embedding was compared against a database of stored slide embeddings for quick retrieval of similar cases, thereby enabling pathologists to rapidly find relevant reference slides, accelerating diagnosis or facilitating training^34,35^. This approach was computationally lightweight, with near-instant matches. Qualitative reviews by a board-certified pathologist indicated that the slide search approach yielded highly similar cases both within a given cohort and across multiple cohorts. Interestingly, in a few instances, cases from other entities would be identified (e.g., a BRCA case while looking for a STAD case). This happened, when special subtypes were queried (e.g., a medullary carcinoma, which can occur both in the stomach as well as in the mammary gland) (Fig. 8d). This could prove to be a valuable tool, for example for cohort assembly for basket trials.

Together, these data indicate that EAGLE enables more transparent slide-level decision-making and simplifies downstream analyses such as multi-omics and clinical integration, and efficient retrieval of relevant comparator slides (Fig. 1d).

### Comparison with multimodal large language models (MLLMs)

Finally, we compared EAGLE with emerging MLLMs employing in-context learning low-resolution WSI thumbnails. Using *k* = 2 examples per class in a few-shot classification setup, EAGLE embeddings combined with a lightweight logistic regression model surpassed GPT-4o’s in-context classification for tasks including NSCLC subtyping, MSI prediction in CRC, and estrogen receptor (ER) expression in BRCA. In NSCLC subtyping and ER expression prediction, GPT-4o usually defaulted to a single class (e.g., predicting adenocarcinoma in NSCLC and ER-positive in BRCA), while in all three tasks, including MSI prediction, its classification accuracy remained at chance level (0.5). To evaluate whether input resolution constrained GPT-4o’s performance, we provided the model with EAGLE’s top 25 most informative tiles instead of WSI thumbnails. Despite this targeted input, MLLM accuracy remained unchanged, suggesting that the bottleneck lies in the models’ lack of pathology-specific feature representations rather than the resolution or relevance of the input (Fig. 2e, Supplementary Fig. 17a). Interestingly, despite its poor predictive accuracy, GPT-4o’s textual responses frequently referenced key diagnostic features, such as “mucin production” and “keratinization” for NSCLC subtyping, “lymphocytic infiltration” for MSI status prediction, indicating an awareness of relevant pathological concepts (Supplementary Fig. 17b).

Taken together, these results underscore the limitations of general-purpose MLLMs in specialized pathology tasks and highlight the need for domain-adapted models. While MLLMs such as GPT-4o demonstrate impressive contextual reasoning and linguistic capabilities, their ability to generalize to specialized pathology biomarker tasks is notably limited.

## Discussion

Weakly supervised CPath has fundamentally reshaped cancer research, allowing diagnosis, biomarker status prediction, and survival outcome estimation directly from WSIs. Recent progress with pathology foundation models, often trained using SSL, has enriched these pipelines, enhancing accuracy and generalizability across diverse clinical tasks. However, concerns remain about the scalability of tile-level methods and the difficulty of interpreting predictions when massive neural networks process thousands of patches per slide. Here, we introduced EAGLE, a slide-level framework that emulates how pathologists target limited, high-yield regions.

EAGLE’s design centers on processing only 25 tiles per WSI, a strategy that substantially reduces computational requirements while preserving predictive power. This design is not a heuristic truncation but reflects a bias–variance trade-off under weak supervision: when the predictive signal is spatially sparse relative to total tissue area, restricting inference to a reproducible, high-saliency subset can improve statistical conditioning. This approach aligns with established clinical practice, in which pathologists focus on diagnostically relevant or suspicious tissue regions rather than exhaustively scanning each tile. Our results show that EAGLE substantially reduces feature extraction times compared to tile-level pipelines relying on resource-intensive foundation models. These efficiency gains translate to quicker classifier training and inference, making EAGLE especially suited for large-scale or multi-institutional datasets. Such performance improvements are likely to be important for real-time or near-real-time diagnostic workflows. By substantially reducing computational requirements, EAGLE brings the deployment of digital pathology tools on lower-resource hardware, including tablets and potentially smartphones, within reach. This improved efficiency broadens accessibility, lowers infrastructure barriers and supports adoption in a wider range of clinical and resource-limited settings.

Our comparative evaluations indicate that EAGLE frequently outperforms or matches leading slide- and tile-encoder baselines, particularly in biomarker prediction tasks central to precision oncology. Whereas many CPath models demonstrate high performance for commonly studied tasks such as tumor subtyping, they can struggle in more challenging settings like molecular status prediction for biomarkers or survival analyses. EAGLE addresses these obstacles by focusing on the regions that matter most morphologically, offering refined predictive performance. Our benchmarking across 31 externally validated tasks shows that combining CHIEF and Virchow2 in EAGLE outperforms both individual models in the majority of cases and is never worse than both on any task. Targeted selection of informative tiles proved essential for maximizing accuracy in our benchmark, whereas selecting more than 25 tiles tended to introduce noise and reduce performance. The strong performance of a fixed 25-tile budget across diverse endpoints should therefore be interpreted as a robust empirical operating point rather than as a biologically universal optimum. Histopathology contains substantial spatial redundancy, and CHIEF ranking appears to recover much of the predictive signal early. However, this advantage is task-dependent: for morphology-heavy endpoints that require broader architectural context or longer-range spatial dependencies, dense slide encoders can remain competitive or superior, as illustrated by TITAN in non-small cell lung cancer subtyping. More generally, any sparse-sampling strategy carries a residual risk of missing rare, spatially dispersed, or globally contextual cues that a dense whole-slide encoder or full human review could capture.

The strength of this approach also reflects the extensive pretraining of CHIEF on more than 60,000 slides, a scale not feasible for typical task-specific datasets in digital pathology. Importantly, performance gains cannot be attributed to arbitrary subsampling. Across multiple tile budgets, saliency-guided selection consistently outperformed repeated uniform random selection under identical splits, indicating that the improvement arises from structured region ranking rather than from processing fewer tiles. Quantitative analysis of attention concentration further showed that the pretrained saliency prior induces a stable and non-uniform ordering of tissue regions, whereas task-specific attention learning frequently approaches near-uniform aggregation for subtle biomarker endpoints. Across models, extensive hyperparameter tuning provided only negligible benefit on external test cohorts, suggesting that performance in large embedding-based CPath models is primarily determined by representation quality rather than classifier optimization. Beyond the benchmarking study, EAGLE maintained strong performance on the PathoBench survival and treatment response tasks, demonstrating its applicability to more complex clinical endpoints.

A hallmark of EAGLE’s design is the generation of a single embedding per slide or patient. Although this embedding remains abstract, EAGLE provides explicit spatial localization because it is computed solely from the features of the top selected tiles, all of which contribute equally to the prediction. While sparsity alone does not guarantee faithful explanation, this design provides strong auditability: the exact regions used for each prediction can be enumerated, reproduced, and rapidly reviewed. In contrast, standard attention heatmaps can be diffuse or unstable across folds. Deep learning systems are often perceived as “black boxes” with limited transparency in medical settings; in contrast, EAGLE allows rapid review of the exact regions used for representation construction. In our qualitative assessment, selected tiles frequently corresponded to plausible tumor-rich regions and showed reduced artifact exposure^36^. Beyond improving interpretability, the resulting slide- and patient-level embeddings open new possibilities for content-based slide retrieval, clustering, and integration with other data modalities. By compressing the most relevant morphological cues into a single vector, EAGLE provides an efficient bridge to genomic, transcriptomic, and radiologic data. In the long term, such patient-level embeddings could serve as the backbone of comprehensive multimodal models, enabling more holistic characterizations of disease states. The field of CPath is expanding beyond image-only pipelines into vision-language models or MLLMs that can annotate slides in a chatbot-like manner, but reveal significant limitations in specialized tasks. Despite referencing key diagnostic features, GPT-4o fails to achieve accuracy beyond chance levels in biomarker prediction, even when provided with EAGLE’s selected tiles. While MLLMs may complement traditional approaches in interpretability, their predictive capacity in pathology remains limited without domain adaptation.

Although we tested EAGLE extensively on multiple external cohorts spanning nine major cancer types, additional large-scale validation is warranted to assess its performance on rare pathologies. Encouragingly, recent studies have demonstrated that large-scale pathology foundation models can generalize well to rare and underrepresented cancer types, supporting the promise of such approaches for broader clinical use^7^. Furthermore, our approach may be less effective for tasks that depend on very specific tissue areas, such as vascular invasion, or on highly heterogeneous tumors composed of multiple distinct subtypes. CHIEF was primarily pre-trained on cancer slides, so its region selection may be less effective for non-cancer tasks, thereby impairing EAGLE’s performance. A limitation of our interpretability analysis is that pathologist review was limited to a single expert and not all methods were assessed in parallel. Future studies should involve multiple pathologists and a standardized evaluation protocol. Our study prioritizes benchmarking predictive performance over detailed confounder analysis, as we focus on common predictive targets to ensure comparability across models. While this facilitates systematic evaluation, it does not capture the influence of confounding variables or the deeper morphological basis of predictions. As training datasets primarily originate from major cancer centers, they may not fully reflect the diversity of global patient populations, highlighting the need for broader validation across more heterogeneous cohorts. While EAGLE achieved AUROCs >0.900 for some tasks, the average AUROC of 0.742 indicates that EAGLE’s performance might not yet be adequate to replace standard clinical procedures. Future work may explore prospective clinical trials to evaluate real-world diagnostic improvements, as well as the integration of EAGLE’s embeddings with patient-specific data, such as laboratory values or electronic health records.

In conclusion, EAGLE demonstrates that combining complementary pathology foundation models can yield representations that are both efficient and broadly generalizable. By integrating large-scale pretrained knowledge through targeted region selection, EAGLE establishes an effective paradigm for building high-performing slide-level models without requiring additional large-scale data collection.

## Methods

### Ethics statement

This study complies with all relevant ethical regulations and was conducted in accordance with the Declaration of Helsinki. TCGA and CPTAC comprise retrospective, de-identified public resources and did not require additional ethics approval for the present secondary analysis. The DACHS study is an epidemiological study overseen by the German Cancer Research Center (DKFZ, Heidelberg, Germany). It was approved by the Ethics Committee of the Medical Faculty of Heidelberg University (310/2001) and the state medical boards of Baden-Württemberg (M-198-02) and Rhineland-Palatinate (837.419.02 [3637]); all participants provided written informed consent^37–39^. The Kiel cohort was approved by the Ethics Committee of the University Hospital Schleswig-Holstein, Campus Kiel (D 453/10), and comprised pseudonymized samples from patients who had provided written informed consent for scientific use. The Bern cohort was approved by the Cantonal Ethics Commission of the Canton of Bern (KEK 200/14). Written informed consent was waived because the study retrospectively reused residual archival tissue collected during routine clinical care, a substantial proportion of patients were already deceased, and recontacting patients or their relatives was considered impracticable and disproportionate. Patients with a documented objection to the use of their tissue or data for research were excluded. The IEO cohort was used under approval of the Institutional Review Board of IEO Milan. The requirement for informed consent was waived because the study retrospectively used archival materials and no directly identifiable patient data were processed. The GECCO data used here derive from previously approved contributing studies coordinated through Fred Hutchinson Cancer Center and are governed by the ethics approvals and consent provisions of the respective component studies. All contributing studies obtained written informed consent from all participants and received approval from their respective institutional review boards. The harmonized, deidentified data were used in accordance with the applicable study approvals and consortium data-use requirements.

### Datasets

This study benchmarks prediction tasks spanning nine major cancer types—breast, colorectal, stomach, lung, prostate, ovary, head and neck, kidney and pancreas—across a range of clinically relevant endpoints, including tumor morphology, molecular biomarker status, treatment response and prognosis. All histopathology data consist of hematoxylin and eosin (H&E)-stained WSI, including both formalin-fixed paraffin-embedded and fresh frozen tissue. Cohorts span a range of staining protocols and scanner hardware, representing the variability encountered in real-world clinical practice. Our evaluation leverages large, multi-institutional datasets for both training and independent external validation, ensuring robust and generalizable performance assessment. High-level summaries of all training and validation datasets, prediction endpoints, and clinical variables are provided in Supplementary Tables 5–7 for reference. All models used in this study, except where explicitly noted, were trained exclusively on TCGA whole-slide images (WSIs), which included histopathology data from lung adenocarcinoma (LUAD), lung squamous cell carcinoma (LUSC), colorectal cancer (CRC), stomach adenocarcinoma (STAD), and breast cancer (BRCA). External validation was carried out on CPTAC (including CPTAC−2 and CPTAC-3 prospective cohorts from 2018 and 2020, covering LUAD, LUSC, COAD, and BRCA), DACHS (CRC), proprietary STAD datasets (Kiel and Bern), and a BRCA dataset (IEO) (Supplementary Fig. 1c). None of these external cohorts were used to pretrain the foundational models or train the downstream classifiers, ensuring that no data leakage occurred. Additional information about the cohorts, including patient characteristics such as age, sex, race, ethnicity, cancer stage, and tumor stage, is provided in Supplementary Table 5. Sex, race, and ethnicity were obtained from the original or harmonized source-cohort metadata where available and were not inferred by the authors. Whether these variables were originally self-reported or abstracted from clinical records was not consistently documented across cohorts. Gender and genetic ancestry were not available as harmonized variables. Sex, race, and ethnicity were reported descriptively and were not used as model inputs, covariates, or prespecified stratification variables. Race and ethnicity were not interpreted as proxies for genetic ancestry.

For the PathoBench dataset, we evaluated 12 prediction tasks encompassing survival and treatment response endpoints across multiple cancer types, including breast, colorectal, ovarian, prostate, head and neck, kidney, and pancreatic cancers. The seven survival tasks comprise one progression-free survival endpoint (BOEHMK, ovarian) and six overall survival endpoints (SURGEN, colorectal; CPTAC-LUAD, lung; CPTAC-HNSC, head and neck; CPTAC-PDA, pancreas; CPTAC-CCRCC, kidney; and MBC, metastatic breast). The five treatment-response tasks included 5-year mortality (SURGEN, colorectal), RECIST response (MBC, breast), lymphovascular invasion (Post-NAT-BRCA, breast), hormonal therapy response (NADT-Prostate, prostate), and bevacizumab response (OV-Bevacizumab, ovarian). In contrast to the main benchmarking framework, PathoBench does not include external validation cohorts; all results were obtained using the predefined internal cross-validation splits provided with the dataset. All histopathology data consisted of H&E-stained slides from multiple institutions, following the standardized preprocessing and evaluation procedures defined in the PathoBench protocol^31,32^.

For biomarker discovery experiments (results presented in Fig. 7f, Supplementary Table 4), an additional dataset from the Genetics and Epidemiology of CRC Consortium (GECCO) was employed, comprising five studies: CORSA, EPIC, CRA, WHI, and IWHS^40,41^ (Supplementary Fig. 1d, Supplementary Table 6). To ensure a minimum of 10 cases per class for both training and testing while maximizing the inclusion of biomarkers, different training and testing splits were used for each biomarker. Three centers were selected for training and two for testing, with the specific splits detailed in Supplementary Table 4 for biomarkers achieving AUROC > 0.800.

### Experimental design

Our study emulates a recently established large-scale benchmarking study^22^. To reflect a broad range of tasks relevant in digital pathology, three main categories were defined, namely morphological, biomarker, and prognostic tasks. Morphological tasks involved the classification of CRC slides as left or right colon (excluding transverse colon), STAD slides into Lauren subtypes, and NSCLC slides into adenocarcinoma (LUAD) or squamous cell carcinoma (LUSC). Biomarker tasks targeted molecular or expression features such as *BRAF*, *KRAS*, *PIK3CA* mutation, microsatellite instability (MSI) and CpG island methylator phenotype (CIMP) status in CRC, Epstein–Barr virus (EBV) and MSI status in STAD, *EGFR*, *STK11*, *KRAS* and *TP53* mutation in LUAD, and human epidermal growth factor receptor 2 (HER2), ER, progesterone receptor (PR) expression, and *PIK3CA* mutation in BRCA. Prognostic tasks addressed nodal involvement (N vs. N0) and metastasis (M0 vs. M+). We included only tasks with at least ten available cases per label in both training and test cohorts. In total, 31 tasks were used, and all were binary except Lauren classification (Supplementary Table 7).

The classifiers were trained and evaluated using a five-fold split of the TCGA data: in each fold, 80% of the data was used for training and 20% for validation (to perform early stopping). This yielded five independently trained models per task, each trained on a different partition of the training sets. Each of these five models was then applied to the external test cohorts (CPTAC, DACHS, Kiel, Bern, IEO) and the results were averaged to provide a robust estimate of performance while minimizing single-run bias. This improves statistical validity and ensures external validation by testing exclusively on datasets not used during training. Performance metrics included AUROC, AUPRC, Balanced Accuracy, and F1 Scores and always represent the mean across the five folds.

We followed the PathoBench protocol and used the predefined eighty percent train and twenty percent test splits. Within each training split, we defined a validation subset comprising twenty percent of cases at the case level. For categorical endpoints we stratified the validation selection when feasible. The test portion was never used for model selection. PathoBench supports early fusion of multiple slides per patient for models that do not rely on spatial coordinates. Consequently, TITAN and GigaPath were evaluated by pooling each slide separately and averaging slide embeddings to obtain a patient representation, whereas all other models used early fusion to form a single patient-level bag prior to pooling. For survival endpoints we trained CoxNet on patient-level embeddings and selected hyperparameters on the validation subset. We searched alpha values of 0.01, 0.05, and 0.1 and L1 mixing ratios of 0.1, 0.5, and 0.9, choosing the configuration that maximized the validation concordance index before evaluating on the held out test set. Survival tasks used five-fold cross validation. For treatment response endpoints we used a linear probing classifier with a single regularization parameter. We searched *C* equal to 0.001, 0.01, 0.1, 0.5, 1.0, and 10, selecting the value that maximized validation AUROC before evaluating on the test set. The fold schedule followed PathoBench: SURGEN 5 y death used five-fold cross validation, whereas MBC RECIST, NATBRCA L. inv., NADT response, and OVARIAN Response used fifty fold Monte Carlo cross validation. Results are reported as mean ± standard error across folds, following the PathoBench protocol. To ensure comparability across methods, all models used the same predefined splits, the same train and validation construction, and the same early versus late fusion setting as described above. PathoBench contains no external cohorts, so all PathoBench results reflect internal cross validation.

### Image processing and deep learning techniques

We adopted the STAMP pipeline (see “Code availability”) for WSI segmentation, tile extraction, and subsequent feature generation. Additional software packages and version numbers used for data analysis are listed in the Reporting Summary. STAMP (Solid Tumor Associative Modeling in Pathology) is an open-source protocol and software for deep learning-based biomarker prediction from whole-slide images, enabling end-to-end modeling and integration of clinical and tabular data in CPath workflows^18^. WSIs were divided into patches (tiles) of size 224 × 224 pixels (or 512 × 512 pixels for CONCH v1.5/TITAN, downsampled to 448 × 448 pixels before model inference). The final effective resolution could be 0.5, ~1.14, or 2 MPP, depending on the foundation model’s magnification preference. For CONCH v1.5, due to downsampling, the effective resolutions were ~0.57 and ~2.28 MPP. Each combination of model and resolution was evaluated, with the best magnification reported in the main experiments. Tiles predominantly containing background were excluded using Canny edge detection (thresholds: 40, 100), rejecting those with fewer than 2% of pixels classified as edges^42^. Before model input, all images were normalized according to the requirements of each foundation model: for CHIEF and Virchow2, standard ImageNet mean and standard deviation values ([0.485, 0.456, 0.406] and [0.229, 0.224, 0.225]) were used, while other models used their respective recommended normalization statistics. No dedicated stain normalization was applied. Remaining tiles were passed into a tile-level foundation model to generate feature embeddings, ranging in dimensionality from 512 (CONCH) to 1536 (Prov-GigaPath), forming an *N* × *M* matrix of tile features per WSI, where *N* is the number of tiles and *M* is the feature dimensionality.

Two main approaches were taken to obtain slide-level representations. In the supervised approach, tile embeddings were fed into a task-specific aggregator, typically a transformer-based network (STAMP) or attention-based multiple instance learning (ABMIL), in order to produce supervised predictions for each classification endpoint^18,21^. In this scenario, every new task required retraining the aggregator model. We evaluated two predefined attention-based MIL baselines: standard ABMIL^21^ and a gated attention variant^21^. For both, hyperparameters were fixed a priori and applied uniformly across all tasks without task-specific tuning. The standard ABMIL configuration followed a commonly used setting with single-head attention and bag size 512. The gated ABMIL baseline was configured to avoid potential undercapacity effects by using full bags (no tile subsampling) and multiplicative gating in the attention pathway. Detailed configurations are provided in Supplementary Table 8. Recently, slide encoders were introduced, which combine tile embeddings (and sometimes tile coordinates) into a single embedding vector for each slide or patient. This strategy is agnostic to the specific task and thus enables a unified, unsupervised representation that can be readily extended to multiple downstream applications by training a small classifier. Detailed hyperparameters for all classifiers are listed in Supplementary Table 8.

EAGLE (Efficient Approach for Guided Local Examination) is a two-stage framework that uses the pre-trained, frozen, task-agnostic ABMIL model CHIEF to select the most informative regions within each WSI, followed by detailed feature extraction from these regions using the state-of-the-art tile encoder Virchow2. CHIEF was pre-trained on over 60,000 whole-slide images spanning 19 anatomical sites. Its pretraining combines tile-level SSL with weakly supervised slide-level contrastive learning and anatomical site encoding, enabling robust region selection across diverse cancer types without the need for retraining. Virchow2 is a state-of-the-art vision transformer trained on over 3 million WSIs from diverse tissues and institutions. The EAGLE framework therefore combines a slide-level foundation model with a tile-level foundation model in a unique way to efficiently generate precise WSI embeddings. EAGLE emulates the pathologist’s workflow by first obtaining an overview of the entire slide, then computationally focusing on the most relevant regions by processing only these with a larger, higher-capacity tile encoder. Since EAGLE requires CTransPath tile embeddings as input, we first extracted features using CTransPath at 2 MPP. CHIEF then uses these embeddings to produce a slide-level representation and an attention vector, originally designed to aggregate tile embeddings into a single slide embedding. EAGLE repurposes this attention vector to identify the top 25 most informative tiles, mimicking how pathologists zoom in on pertinent areas. These tiles are reprocessed with Virchow2 to extract detailed feature embeddings. The resulting 25 embeddings are averaged (i.e., each tile contributes equally) to create a compact, unsupervised slide-level representation. We chose equal weighting to avoid the risk of single-tile dominance and for conceptual simplicity. This hybrid approach minimizes computational costs by applying a more powerful feature extractor only to the most informative tiles, preserving critical morphological details while avoiding unnecessary processing of less relevant regions. The final embeddings enable the use of lightweight models, such as a small MLP classifier, for downstream tasks. This efficient pipeline demonstrates a balance of performance and computational feasibility for large-scale pathology analyses. The EAGLE framework supports replacement of both the tile and slide encoders. We tested substituting Virchow2 with other tile encoders in ablation studies (see Fig. 3b). For the slide encoder, any model that provides tile-level relevance or attention scores could be used in place of CHIEF. Models without explicit tile-level scores are not compatible with this approach. We chose CHIEF specifically for its strong performance and computational efficiency, aligning with the efficiency goals of EAGLE. Negative control experiments for region selection were performed using uniform random tile selection without replacement from the same tile set available to CHIEF for each patient. For each tile budget *N* in [5, 10, 25, 50, 100], we generated *R* = 100 independent random selections per fold and evaluated each replicate under the identical training and testing protocol used for the corresponding CHIEF-selected setting. For each replicate we report the mean AUROC aggregated across all tasks and folds, yielding an empirical null distribution for unguided subsampling.

Multiple other slide encoders and their tile-level foundations were also assessed (Supplementary Tables 9, 10). Prov-GigaPath used a masked autoencoder scheme on 171,189 WSIs from Providence, adopting a LongNet architecture with dilated attention at the slide level and a ViT-G/14 tile encoder trained with DINOv2^9,43^. Prism applied a Perceiver-based architecture with CoCa-style vision-language alignment, trained on 587,196 WSIs in 195,344 specimen-report pairs, and employed a ViT-H/14 tile encoder called Virchow, which was also pretrained with DINOv2^10,26^. CHIEF was trained via slide-level contrastive learning and anatomic site information, using CTransPath with a SwinTransformer architecture as the tile encoder^8,23^. MADELEINE was pretrained on multistain data from breast samples using a dual global/local cross-stain alignment, and it built on CONCH, a vision-language CoCa model with 1.1 million image-text pairs^27,28^. TITAN used a multi-stage pretraining regime combining visual self-supervision, alignment with pathology reports, and 423,122 synthetic captions generated by a multimodal AI copilot; it employed CONCH v1.5 for tile embeddings, relying on UNI as its vision backbone and CoCa’s text tower^7,30,44^. COBRA was trained on 3048 WSIs to align tile embeddings from multiple foundation models via a contrastive loss, yielding a slide-level representation^29^. The best version of COBRA uses Virchow2 features, which expands on Virchow by scaling its dataset to 3.1 million WSIs and using domain-specific modifications of the DINOv2 framework^24^. COBRA and CHIEF were (partly) trained on TCGA, but none of the models used our external testing cohorts, preventing data leakage. We compared each slide encoder against a straightforward mean pooling baseline, where tile embeddings are simply averaged to yield an unsupervised slide-level feature vector. For each slide encoder and for STAMP, we evaluated multiple magnifications (0.5 MPP, 1.14 MPP, 2 MPP) and selected the resolution yielding the best downstream performance for each model and task. For PathoBench tasks, we used the magnification recommended by the original authors. Across all models, performance differences between magnifications were generally minor and no single resolution was consistently optimal.

For certain patients with multiple WSIs (e.g., multiple tissue blocks), each slide encoder was applied in two possible ways. Either each slide was independently processed, and the embeddings were averaged to yield a single patient representation, or all tiles were passed simultaneously into the slide encoder to produce one patient embedding per forward pass. Combining all tiles led to higher performance of all slide encoders, but due to memory constraints on a 48 GB GPU we had to cap the maximum number of features for TITAN at 15,000.

Once a slide-level or patient-level embedding was computed, it was fed into a small multilayer perceptron (MLP) to yield final predictions for each classification task. This MLP used an input size of 768, had a hidden size of 256 with SiLU activation and dropout, and was trained for 32 epochs using a one-cycle policy with the AdamW optimizer (learning rate = 1 × 10^−4^, weight decay = 1 × 10^−2^). Cross-entropy loss with class weighting addressed label imbalance. Early stopping, monitored through validation loss in a five-fold cross-validation scheme, selected the best checkpoints. For few-shot linear probing experiments, we replaced the MLP with a logistic regression model (lbfgs solver, L2 penalty = 1.0, 10,000 maximum iterations, and balanced class weights) and trained on *k* = 1, 2, 4, 8, 16, 32 samples per class (Supplementary Fig. 1a, b, Supplementary Table 6). We repeated each setting ten times with different random draws to stabilize the results. This approach allowed us to evaluate how effectively slide encoders adapted to extremely limited training data.

To compare EAGLE with a MLLM, we tested EAGLE’s few-shot performance (*k* = 2) against GPT-4o in-context learning on three specific tasks: NSCLC subtyping (LUAD vs. LUSC), MSI status prediction in CRC, and ER expression in BRCA. EAGLE used its standard logistic regression approach, while GPT-4o received prompts containing two example images per class, plus the query image (top 25 EAGLE-selected tiles vs. a single thumbnail) (Supplementary Table 11). The GPT-4o model was run three times per image to reduce variability, with temperature = 0.7 and a maximum token length of 1000. A strict JSON format in the prompt forced GPT-4o to provide a single label in each response.

An additional analysis was conducted to examine computational efficiency. We selected 25 representative WSIs (at percentiles 2, 6, 10, …, 98 by tile count) and measured the inference time and floating-point operations (FLOPs) for each tile encoder. FLOP counts were derived using ptflops and were multiplied by the average tile count in the overall dataset to estimate total computational costs.

### Interpretability

To interpret the embedding spaces generated by EAGLE, we applied UMAP dimensionality reduction (n_neighbors = 15, min_dist = 0.1) to project patient embeddings from all cohorts into two dimensions. This visualization enabled the assessment of morphological clustering by tissue origin. Separate experiments tested how EAGLE’s embeddings facilitated slide-level retrieval by normalizing each embedding with L2 and performing a cosine similarity search. We queried five random patients per external cohort and returned the top three matches. The retrieved slides were assessed by a board-certified pathologist regarding the quality and importance of the selected regions of the WSIs.

Attention concentration metrics were computed from the per-tile attention weights produced by CHIEF, ABMIL, and gated ABMIL after softmax normalization within each patient bag. For each patient, tiles were ranked by attention and we computed Lorenz curves as the cumulative attention mass versus the cumulative fraction of tiles. We summarize concentration using the Gini coefficient, the fraction of tiles required to accumulate 50% and 80% of total mass, and top-k mass statistics (top 1, top 2, and top 25). To aggregate Lorenz curves across tasks without averaging attention for the same physical tile across different models or folds, we averaged rank profiles: for each patient we align by rank position (rank 1, rank 2, etc) and then average these ordered attention values across patients and tasks.

Furthermore, to investigate the robustness of EAGLE’s top-tile selection, particularly in the presence of artifacts, we conducted a systematic review of its top 25 tiles on 50 randomly selected slides from the DACHS CRC cohort. A board-certified pathologist reviewed these tiles to determine the frequency of artifact focus (e.g., pen marks) versus tumor-rich regions. This performance was compared to a supervised baseline: Virchow2 tile embeddings were aggregated in STAMP on the MSI status prediction task. We computed Gradient-weighted Class Activation Mapping (Grad-CAM) attribution scores and selected the 25 tiles with the highest scores^45^. A board-certified pathologist analyzed the tiles selected by both EAGLE and STAMP, focusing on their clinical relevance and morphological significance.

For attention map visualizations, per-tile attention values were mapped back to WSI coordinates using the tile grid produced during tessellation. Heatmaps were rendered by assigning each tile its attention value. Unless stated otherwise, heatmap color scales were normalized per panel to visualize each model’s dynamic range. Matched-scale controls are provided in Supplementary Fig. 12.

### Statistical analysis

All classification performance results across tasks were aggregated from the five models produced in each cross-validation fold and summarized via metrics such as mean AUROC, AUPRC, balanced accuracy, and F1. Standard deviations were computed across the five folds, and two-sided DeLong’s tests were applied to ensembled predictions (averaging fold probability scores for each sample) to assess differences in AUROC. Multi-class tasks like Lauren classification were excluded from these statistical tests because DeLong’s procedure is not directly applicable in the multi-class setting. Throughout, we used Benjamini–Hochberg adjustments and considered *p*-values below 0.05 as evidence of statistical difference. We used the c-index to evaluate the performance of the prognostic models for survival endpoints. Kaplan–Meier curves were generated to assess patient stratification, using the median predicted risk score as the cut-off value. The statistical significance of low-risk and high-risk patient groups was assessed using the log-rank test. For negative control experiments with *R* = 100 repeated uniform random tile selection, one-sided Monte Carlo *p*-values were computed as (*r* + 1)/(*R* + 1), where *r* is the number of random replicates that meet or exceed the CHIEF-selected result.

### Reporting summary

Further information on research design is available in the Nature Portfolio Reporting Summary linked to this article.

## Supplementary information

Supplementary Information File

Reporting Summary

Transparent Peer Review file

## Source data

Source Data

## Data Availability

WSIs from TCGA are publicly available through the Genomic Data Commons Data Portal (https://portal.gdc.cancer.gov/). CPTAC data are publicly available through the National Cancer Institute CPTAC resources and associated data portals (https://proteomics.cancer.gov/data-portal). Molecular data for TCGA and CPTAC can be accessed through cBioPortal (https://www.cbioportal.org/). The PathoBench dataset is publicly available on Hugging Face (https://huggingface.co/datasets/MahmoodLab/Patho-Bench). Patient-level data from DACHS, Kiel, Bern, IEO, and GECCO are third-party clinical datasets and are not publicly deposited because redistribution is constrained by the original ethics approvals, consent conditions, applicable privacy law, and institutional or consortium data-use and transfer agreements. The restricted data comprise H&E whole-slide images and linked clinicopathological, biomarker, and/or molecular variables used in this study. Access is limited to qualified researchers acting through institutions that can enter the required agreements, and proposed use must be compatible with the approved scientific purpose and any cohort-specific data-use limitations. Initial responses to data access requests can generally be expected within several weeks; the total time to execute a data use or transfer agreement may be longer depending on institutional review processes at the requesting and data-holding institutions. Access duration is determined case by case by the data holder during review and contracting. The slides and biomarker data for DACHS were generated for prior studies^46–48^ with restricted access. DACHS biomarker and genotype data can be requested through dbGaP Authorized Access via the GECCO top-level study phs001078, with DACHS represented as sub-study phs001113.v1.p1 [https://www.ncbi.nlm.nih.gov/projects/gap/cgi-bin/study.cgi?study_id=phs001113.v1.p1]. Applications for access to DACHS biomarker data are reserved for Senior Investigators and NIH Investigators as defined in https://dbgap.ncbi.nlm.nih.gov/aa/wga.cgi, and upon successful application grants access to the data for 1 year with the option to renew access. The slides for DACHS can only be requested directly through the DACHS principal investigators. The contact details are listed at http://dachs.dkfz.org/dachs/kontakt.html. Kiel WSIs and linked clinicopathological data are held by the Department of Pathology, University Hospital Schleswig-Holstein, Kiel, Germany. Requests should be directed to the department through its official contact page (https://www.medizin.uni-kiel.de/en/institutes-departments/institutes-of-clinical-theory/department-of-pathology). Bern whole-slide and linked clinicopathological data are held by the Institute of Tissue Medicine and Pathology, University of Bern, Switzerland; individual patient-level data are not publicly shared, and requests should be directed to the institute (contact.igmp@unibe.ch) in reference to ref. ^49^. IEO whole-slide and linked clinicopathological data are held by the European Institute of Oncology, Milan, Italy; requests are evaluated case by case under institutional policies and patient-privacy obligations and should be submitted through the institute’s official contact route (https://www.ieo.it/en/contact_us/). GECCO H&E WSIs and associated clinicopathological and molecular data used in this study are coordinated through the GECCO consortium at Fred Hutchinson Cancer Center; requests should be directed to the GECCO coordinating center (gecco@fredhutch.org) and may require approval consistent with the policies of the contributing studies (CORSA, EPIC, CRA, WHI, IWHS). Source data are provided with this paper.

## Code availability

All benchmarking experiments build upon the open-source STAMP-Benchmark software, available at https://github.com/KatherLab/STAMP-Benchmark and archived at Zenodo under DOI 10.5281/zenodo.15749283 (ref. ^50^), released under the MIT License. The implementation of EAGLE, including the tested slide encoders, the MLP and linear-probing classifiers, the GPT-4o in-context-learning evaluation, UMAP visualization, top-tile extraction, and slide-search analyses, is available at https://github.com/KatherLab/EAGLE and is archived at Zenodo under 10.5281/zenodo.19799127 (ref. ^51^), released under the GNU General Public License v3.0 (GPL-3.0). The repository includes the software license and citation metadata. Third-party foundation models used in this study are distributed under their respective licenses and access conditions. All experiments were conducted using NVIDIA RTX A6000, L40 or H100 GPUs.
