# Supplementary material for: A deep learning framework for efficient pathology image analysis
Source: Nat Commun. 2026 Jul 1;17:5740. doi: 10.1038/s41467-026-74918-9 (PMC13324285; doi:10.1038/s41467-026-74918-9)
Supplement: Supplementary file 2 — Reporting Summary [file 41467_2026_74918_MOESM2_ESM.pdf]

nature portfolio

Corresponding author(s): Jakob Nikolas Kather

Last updated by author(s): Jun 7, 2026

Reporting Summary

Nature Portfolio wishes to improve the reproducibility of the work that we publish. This form provides structure for consistency and transparency in reporting. For further information on Nature Portfolio policies, see our Editorial Policies and the Editorial Policy Checklist.

Statistics

For all statistical analyses, confirm that the following items are present in the figure legend, table legend, main text, or Methods section.

n/a	Confirmed	
☐	☒	The exact sample size (*n*) for each experimental group/condition, given as a discrete number and unit of measurement
☐	☒	A statement on whether measurements were taken from distinct samples or whether the same sample was measured repeatedly
☐	☒	The statistical test(s) used AND whether they are one- or two-sided<br>*Only common tests should be described solely by name; describe more complex techniques in the Methods section.*
☒	☐	A description of all covariates tested
☐	☒	A description of any assumptions or corrections, such as tests of normality and adjustment for multiple comparisons
☐	☒	A full description of the statistical parameters including central tendency (e.g. means) or other basic estimates (e.g. regression coefficient) AND variation (e.g. standard deviation) or associated estimates of uncertainty (e.g. confidence intervals)
☐	☒	For null hypothesis testing, the test statistic (e.g. *F*, *t*, *r*) with confidence intervals, effect sizes, degrees of freedom and *P* value noted<br>*Give P values as exact values whenever suitable.*
☒	☐	For Bayesian analysis, information on the choice of priors and Markov chain Monte Carlo settings
☒	☐	For hierarchical and complex designs, identification of the appropriate level for tests and full reporting of outcomes
☒	☐	Estimates of effect sizes (e.g. Cohen's *d*, Pearson's *r*), indicating how they were calculated

Our web collection on statistics for biologists contains articles on many of the points above.

Software and code

Policy information about availability of computer code

Data collection	No primary data were collected for this study. Whole-slide images and associated molecular or clinical data were obtained from existing public and restricted-access cohorts as described in the Data Availability statement.
Data analysis	Data analysis was performed using STAMP-Benchmark v1.0.0, archived at Zenodo under DOI 10.5281/zenodo.15749283, together with the EAGLE codebase archived under DOI 10.5281/zenodo.19799127. Core software packages included Python 3.11, PyTorch 2.2, fastai 2.7, scikit-learn 1.3, pandas 2.1, and h5py 3.10. Additional packages used for specific analyses included OpenSlide-Python 1.3 and OpenCV 4.8 for whole-slide image processing, matplotlib 3.8 for figure generation, OpenAI Python 1.59.6 for GPT-4o evaluation, ptflops 0.7.2.1 for FLOP estimation, and umap-learn 0.5.6 for UMAP analyses.

For manuscripts utilizing custom algorithms or software that are central to the research but not yet described in published literature, software must be made available to editors and reviewers. We strongly encourage code deposition in a community repository (e.g. GitHub). See the Nature Portfolio guidelines for submitting code & software for further information.

Data

Policy information about availability of data

All manuscripts must include a data availability statement. This statement should provide the following information, where applicable:

- Accession codes, unique identifiers, or web links for publicly available datasets
- A description of any restrictions on data availability
- For clinical datasets or third party data, please ensure that the statement adheres to our policy

WSIs from TCGA are publicly available through the Genomic Data Commons Data Portal (https://portal.gdc.cancer.gov/). CPTAC data are publicly available through the National Cancer Institute CPTAC resources and associated data portals (https://proteomics.cancer.gov/data-portal). Molecular data for TCGA and CPTAC can be accessed through cBioPortal (https://www.cbioportal.org/). The PathoBench dataset is publicly available on Hugging Face (https://huggingface.co/datasets/MahmoodLab/Patho-Bench). Patient-level data from DACHS, Kiel, Bern, IEO, and GECCO are third-party clinical datasets and are not publicly deposited because redistribution is constrained by the original ethics approvals, consent conditions, applicable privacy law, and institutional or consortium data-use and transfer agreements. The restricted data comprise H&E whole-slide images and linked clinicopathological, biomarker, and/or molecular variables used in this study. Access is limited to qualified researchers acting through institutions that can enter the required agreements, and proposed use must be compatible with the approved scientific purpose and any cohort-specific data-use limitations. Initial responses to data access requests can generally be expected within several weeks; the total time to execute a data use or transfer agreement may be longer depending on institutional review processes at the requesting and data-holding institutions. Access duration is determined case by case by the data holder during review and contracting. The slides and biomarker data for DACHS were generated for prior studies46–48 with restricted access. DACHS biomarker and genotype data can be requested through dbGaP Authorized Access via the GECCO top-level study phs001078, with DACHS represented as sub-study phs001113.v1.p1 [https://www.ncbi.nlm.nih.gov/projects/gap/cgi-bin/study.cgi?study_id=phs001113.v1.p1]. Applications for access to DACHS biomarker data are reserved for Senior Investigators and NIH Investigators as defined in https://dbgap.ncbi.nlm.nih.gov/aa/wga.cgi, and upon successful application grants access to the data for 1 year with the option to renew access. The slides for DACHS can only be requested directly through the DACHS principal investigators. The contact details are listed at http://dachs.dkfz.org/dachs/kontakt.html. Kiel WSIs and linked clinicopathological data are held by the Department of Pathology, University Hospital Schleswig-Holstein, Kiel, Germany. Requests should be directed to the department through its official contact page (https://www.medizin.uni-kiel.de/en/institutes-departments/institutes-of-clinical-theory/department-of-pathology). Bern whole-slide and linked clinicopathological data are held by the Institute of Tissue Medicine and Pathology, University of Bern, Switzerland; individual patient-level data are not publicly shared, and requests should be directed to the institute (contact.igmp@unibe.ch) in reference to ref.49. IEO whole-slide and linked clinicopathological data are held by the European Institute of Oncology, Milan, Italy; requests are evaluated case by case under institutional policies and patient-privacy obligations and should be submitted through the institute's official contact route (https://www.ieo.it/en/contact_us/). GECCO H&E WSIs and associated clinicopathological and molecular data used in this study are coordinated through the GECCO consortium at Fred Hutchinson Cancer Center; requests should be directed to the GECCO coordinating center (gecco@fredhutch.org) and may require approval consistent with the policies of the contributing studies (CORSA, EPIC, CRA, WHI, IWHS). Source data are provided with this paper.

Research involving human participants, their data, or biological material

Policy information about studies with human participants or human data. See also policy information about sex, gender (identity/presentation), and sexual orientation and race, ethnicity and racism.

Reporting on sex and gender	Sex was obtained from the original or harmonized cohort metadata where available and was not inferred by the authors. The original method of ascertainment, including whether sex was self-reported or abstracted from clinical records, was not consistently documented across the contributing cohorts. Gender was not available as a harmonized variable. Sex was reported descriptively and was not used as a model input or covariate. Because this study focused on benchmarking across heterogeneous retrospective datasets and complete harmonized sex and gender metadata were not available across all cohorts, no prespecified sex- or gender-stratified analyses were performed. Cohort-specific availability is summarized in Supplementary Tables 5 and 6.
Reporting on race, ethnicity, or other socially relevant groupings	The cohorts included in this study originate from the following countries: TCGA and CPTAC (USA), GECCO (Europe & USA), DACHS and Kiel (Germany), Bern (Switzerland), and IEO (Italy). Race and ethnicity were obtained from the original or harmonized cohort metadata where available and were not inferred by the authors from histopathology images, participant names, geographic location, or other participant characteristics. For TCGA and CPTAC, race and ethnicity were obtained from clinical metadata available through cBioPortal and originated from data submitted by the contributing institutions. For GECCO, race was available, whereas ethnicity was not available. Race and ethnicity were not available for DACHS, Bern, Kiel, or IEO. The original method of ascertainment, including whether these characteristics were self-reported or abstracted from clinical records, was not consistently documented across the contributing cohorts. Genetic ancestry was not available as a harmonized variable. Race and ethnicity were reported descriptively, were not used as model inputs, covariates, or prespecified stratification variables, and were not interpreted as proxies for genetic ancestry. Cohort-specific information is summarized in Supplementary Tables 5 and 6.
Population characteristics	Available participant characteristics, including cohort size, age, sex, race, ethnicity, cancer stage, and tumor stage, are summarized by cohort in Supplementary Tables 5 and 6. The availability of these characteristics differed among cohorts.
Recruitment	No participants were recruited specifically for the present study. This work is a retrospective secondary analysis of previously collected cohort datasets.
Ethics oversight	This study complies with all relevant ethical regulations and was conducted in accordance with the Declaration of Helsinki. TCGA and CPTAC comprise retrospective, de-identified public resources and did not require additional ethics approval for the present secondary analysis. The DACHS study is an epidemiological study overseen by the German Cancer Research Center (DKFZ, Heidelberg, Germany). It was approved by the Ethics Committee of the Medical Faculty of Heidelberg University (310/2001) and the state medical boards of Baden-Württemberg (M-198-02) and Rhineland-Palatinate (837.419.02 [3637]); all participants provided written informed consent37–39. The Kiel cohort was approved by the Ethics Committee of the University Hospital Schleswig-Holstein, Campus Kiel (D 453/10), and comprised pseudonymized samples from patients who

had provided written informed consent for scientific use. The Bern cohort was approved by the Cantonal Ethics Commission of the Canton of Bern (KEK 200/14). Written informed consent was waived because the study retrospectively reused residual archival tissue collected during routine clinical care, a substantial proportion of patients were already deceased, and recontacting patients or their relatives was considered impracticable and disproportionate. Patients with a documented objection to the use of their tissue or data for research were excluded. The IEO cohort was used under approval of the Institutional Review Board of IEO Milan. The requirement for informed consent was waived because the study retrospectively used archival materials and no directly identifiable patient data were processed. The GECCO data used here derive from previously approved contributing studies coordinated through Fred Hutchinson Cancer Center and are governed by the ethics approvals and consent provisions of the respective component studies. All contributing studies obtained written informed consent from all participants and received approval from their respective institutional review boards. The harmonized, deidentified data were used in accordance with the applicable study approvals and consortium data-use requirements.

Note that full information on the approval of the study protocol must also be provided in the manuscript.

Field-specific reporting

Please select the one below that is the best fit for your research. If you are not sure, read the appropriate sections before making your selection.

☒ Life sciences ☐ Behavioural & social sciences ☐ Ecological, evolutionary & environmental sciences

For a reference copy of the document with all sections, see nature.com/documents/nr-reporting-summary-flat.pdf

Life sciences study design

All studies must disclose on these points even when the disclosure is negative.

Sample size	No explicit sample-size calculation was performed. Recent studies in computational pathology by Foersch et al. (Nat Med, 2023) and Wagner et al. (Cancer Cell, 2023) showed successful biomarker predictions with cohorts having several hundred patients, with larger cohorts yielding better results. Consequently, we collected datasets which satisfied this volume range of patients, having several hundreds, or thousands of patients available for retrospective analysis. Moreover, a recent Nature Protocols Paper by El Nahhas et al. provided rough guidelines for an estimation of sample sizes for computational pathology (https://www.nature.com/articles/s41596-024-01047-2).
Data exclusions	In all experiments, data samples were excluded when the microns-per-pixel information was not available in the metadata of the whole-slide image, or if the biomarker to be predicted was not available for the sample.
Replication	The reproducibility of experimental findings was verified through a 5-fold cross-validation setup, with performance metrics reported as the mean across folds to ensure statistical reliability. External validation was conducted on independent cohorts not used during training, further supporting reproducibility.
Randomization	TCGA data was exclusively used for training to avoid data leakage, as it was part of the pretraining of some of the tested foundation models. In the cross-validation setup, patients within the TCGA cohort were randomly assigned to training and validation folds using a stratified k-fold approach. This ensured that class distributions were balanced across folds, reducing bias and maintaining the robustness of the analysis.
Blinding	This study was conducted retrospectively. Therefore, investigators were not blinded to allocation during experiments or outcome assessment. The data was randomly split into training and validation sets for the training cohort (TCGA), while all other cohorts were used exclusively for external validation. Given this design, blinding was not applicable.

Reporting for specific materials, systems and methods

We require information from authors about some types of materials, experimental systems and methods used in many studies. Here, indicate whether each material, system or method listed is relevant to your study. If you are not sure if a list item applies to your research, read the appropriate section before selecting a response.

Materials & experimental systems

n/a	Involved in the study
☒	☐ Antibodies
☒	☐ Eukaryotic cell lines
☒	☐ Palaeontology and archaeology
☒	☐ Animals and other organisms
☒	☐ Clinical data
☒	☐ Dual use research of concern
☒	☐ Plants

Methods

n/a	Involved in the study
☒	☐ ChIP-seq
☒	☐ Flow cytometry
☒	☐ MRI-based neuroimaging

Plants

Seed stocks	*Report on the source of all seed stocks or other plant material used. If applicable, state the seed stock centre and catalogue number. If plant specimens were collected from the field, describe the collection location, date and sampling procedures.*
Novel plant genotypes	*Describe the methods by which all novel plant genotypes were produced. This includes those generated by transgenic approaches, gene editing, chemical/radiation-based mutagenesis and hybridization. For transgenic lines, describe the transformation method, the number of independent lines analyzed and the generation upon which experiments were performed. For gene-edited lines, describe the editor used, the endogenous sequence targeted for editing, the targeting guide RNA sequence (if applicable) and how the editor was applied.*
Authentication	*Describe any authentication procedures for each seed stock used or novel genotype generated. Describe any experiments used to assess the effect of a mutation and, where applicable, how potential secondary effects (e.g. second site T-DNA insertions, mosiacism, off-target gene editing) were examined.*
