# Supplementary material for: A deep learning framework for efficient pathology image analysis
Source: Nat Commun. 2026 Jul 1;17:5740. doi: 10.1038/s41467-026-74918-9 (PMC13324285; doi:10.1038/s41467-026-74918-9)
Supplement: Supplementary file 3 — Transparent Peer Review file [file 41467_2026_74918_MOESM3_ESM.pdf]

nature portfolio

Peer Review File

A deep learning framework for efficient pathology image analysis

Corresponding Author: Professor Jakob Kather

This manuscript has been previously reviewed at another journal. This document only contains information relating to versions considered at Nature Communications.

This file contains all reviewer reports in order by version, followed by all author rebuttals in order by version.

Version 0:

Reviewer comments:

Reviewer #1

(Remarks to the Author)
I have read the resubmission carefully, and I still have mixed feelings about the work. Overall, the authors are responsive and have addressed most of my comments on the technical and validation aspects.
However, I am still not convinced that this study brings any fundamental innovation to the field. While reducing the computational burden is useful, I am not entirely convinced that this gain is significant, especially given that the reduction stems from a simplistic reduction of the amount of information processed, rather than from algorithmic or principled innovation.

Intuitively, the contextual information in those patches that are excluded by EAGLE should be useful and contribute to the pattern analysis and downstream tasks. For pathologists, the extent of diseased tissue in the context of normal tissue is important. Therefore, I feel that the exclusion of these patches by EAGLE should probably be justified on a more fundamental basis than purely computational speed and performance.

(Remarks on code availability)

Reviewer #4

(Remarks to the Author)
This work proposes EAGLE, a lightweight aggregation framework that selects only the top 25 tiles using CHIEF's frozen, task-agnostic attention scores, followed by feature extraction (with Virchow2) and simple averaging. While impressive speed-ups and competitive AUROCs are reported, several fundamental concerns raised by reviewers remain inadequately addressed.

1. The core design (fixed, task-agnostic tile selection) requires strong justification.
The central assumption, that a single set of 25 tiles selected via CHIEF's pretrained attention suffices for all downstream tasks, is highly questionable. In practice, biologically relevant regions vary significantly across tasks: for instance, given the same LUAD WSIs, different tasks like EGFR mutation prediction, TP53 status, and NSCLC often rely on distinct morphological patterns (e.g., tumor-stroma interface vs. necrotic core vs. immune infiltrate). Prior works (e.g., ABMIL) explicitly learn task-specific attention because generic "informative" regions are insufficient.
Notably,
- Reviewer #1 explicitly asked for analysis on how weight distributions among the top 25 tiles behave (e.g., are embeddings dominated by 1–2 tiles? What features drive high attention?). The authors only clarified that aggregation is uniform, without providing the distribution of CHIEF's attention used for selection.
- In the original CHIEF paper (Fig. 2b), its attention is broad and diffuse (high scores span >50% of tissue regions on the provided four cases). Selecting only 25 from this large pool is arbitrary: if CHIEF's attention is truly flat over diagnostically relevant regions (as implied), then any reasonable subset, including uniformly sampled tiles, should perform comparably.

Yet the manuscript reports drastically worse performance for random selection (e.g., AUROC drop of ~10%, per Fig. 3c), without reporting confidence interval, despite Reviewer #3 requesting systematic negative controls (100 random replicates). This creates an internal inconsistency: either (i) CHIEF's attention is sharper than disclosed (raising transferability concerns across tasks), or (ii) attention-guided selection does not yield a performance improvement (indicating potentially undertuned baselines, as noted by R2).

2. The claimed superiority over Virchow2+ABMIL lacks a fair or mechanistic explanation.
Reviewers #2 and #3 rightly expressed scepticism: "I really would not have expected this method to outperform ABMIL…", "… (EAGLE) essentially an extreme, thresholded implementation of attention-based method…", "the model itself (ABMIL, etc.) should be quite capable of that (filtering out less informative regions). It shouldn't require the patch selection and encoder to do that."
The authors' rebuttal rests on two weak pillars:
(a) Small-data limitation of supervised ABMIL. They claim ABMIL fails due to limited training data. However, there is still no direct evidence on whether task-agnostic large-scale pretrained attention is better than the task-specific attention in ABMIL. Given the facts that ROIs vary across tasks and the attention module is mainly used for feature aggregation rather than extraction, it is more natural and convincing to use the task-specific attention module.
(b) "Filtering noise improves performance". However, ABMIL aggregators already down-weight noisy tiles via learned attention. If unweighted averaging of CHIEF-selected tiles beats attention-weighted aggregation over all tiles, it implies CHIEF's pretrained attention is more discriminative than learned attention, even on out-of-distribution tasks. This contradicts the results from the original CHIEF paper (Fig. 2b) and demands direct visualization (e.g., attention maps across tasks and WSIs) and comparison (e.g., CHIEF's top-25 tiles regions, CHIEF's attention map visualization, and task-specific attention map visualization), neither of which is provided.

3. Interpretability claims may be overstated and not evidence-backed.
The assertion that "using only 25 tiles inherently improves interpretability" conflates sparsity with faithfulness. A sparse selection is only interpretable if the selected tiles are truly diagnostic, yet evaluation remains qualitative (single pathologist without specific metric) and artifact-focused (pen marks are not equivalent to clinical relevance).

To substantiate EAGLE's contributions, the authors should provide:
1. Robust negative controls: performance of uniform tile selection (25/50/100/…), rather than a single random selection without statistical testing.
2. Attention distribution and stability metrics: Show histograms of CHIEF attention scores and ABMIL attention scores; report % of cases where top-25 tiles dominate for both strategies.
3. Direct visualization and comparison on attention maps: Visualize and compare (i) CHIEF attention heat maps, (ii) ABMIL task-specific attention heat maps, (iii) uniform selection patches location on the heat maps, (vi) CHIEF's top-25 tiles on the heat maps. If these results fail to support the authors' argument, the reliability of EAGLE remains questionable.

(Remarks on code availability)

Version 1:

Reviewer comments:

Reviewer #1

(Remarks to the Author)
The manuscript and the presentation of results have been improved; however, I feel that the fundamental methodology remains largely unchanged.

I am a proponent of sparse decision-making, but sparsity is usually a consequence of limited measurement data. When data are abundant or oversampled, researchers typically sparsify based on solid theoretical frameworks. The approach taken in this work appears purely empirical, lacking a well-established theoretical foundation.

While EAGLE's strategy of analyzing only 25 selected tiles significantly accelerates the workflow, it introduces a latent risk of missing critical clinical information. In complex malignancies, sparse sampling—representing only a fraction of the total tissue—may fail to capture the full diagnostic landscape. This contrasts with standard clinical practice, where pathologists must examine the entire Whole Slide Image (WSI), and often multiple levels or blocks, to ensure a comprehensive diagnosis. Indeed, clinical pathology relies on a holistic evaluation of the tumor microenvironment, including stromal interactions and vascular invasion.

EAGLE's "selective focus" mimics the human eye but lacks the global spatial context provided by dense-sampling models. In cases where diagnostic clues are subtle or scattered, a sparse-sampling approach might overlook information that a full-scale review would catch. Furthermore, despite its superior average performance in tasks requiring minimal morphological information, EAGLE is outperformed by models like TITAN in specific categories—particularly morphology-heavy tasks such as lung cancer subtyping. This suggests that for tasks requiring the integration of macro-scale structural features and long-range spatial dependencies, heavy-weight slide encoders that process the full image still maintain a competitive edge.

(Remarks on code availability)

Reviewer #4

(Remarks to the Author)
The authors provide a concrete analysis of EAHLE’s underlying mechanisms. In particular, the analyses of attention concentration and the attention-map visualizations for ABMIL and EAGLE largely address my concerns regarding the tile-selection strategy.
One minor point still warrants discussion. While the manuscript does not claim that selecting the same 25 tiles is biologically optimal for every task, the results in Supplementary Fig. 3a suggest that this choice performs well across different task types. Although I understand that involving additional pathologists to assess the biological relevance of the selected tiles may be challenging, a discussion of this somewhat counterintuitive observation would be helpful.

(Remarks on code availability)
It looks good to me.

Open Access This Peer Review File is licensed under a Creative Commons Attribution 4.0 International License, which permits use, sharing, adaptation, distribution and reproduction in any medium or format, as long as you give appropriate credit to the original author(s) and the source, provide a link to the Creative Commons license, and indicate if changes were made.
In cases where reviewers are anonymous, credit should be given to 'Anonymous Referee' and the source.
The images or other third party material in this Peer Review File are included in the article’s Creative Commons license, unless indicated otherwise in a credit line to the material. If material is not included in the article’s Creative Commons license and your intended use is not permitted by statutory regulation or exceeds the permitted use, you will need to obtain permission directly from the copyright holder.
To view a copy of this license, visit https://creativecommons.org/licenses/by/4.0/

We thank the reviewers for their careful re-evaluation and for the specific guidance on the key remaining concerns. In this revision we focused on one central question: whether EAGLE's fixed, task-agnostic tile selection is a principled solution to a measurable limitation of end-to-end weak supervision, rather than an arbitrary truncation that happens to be fast.

To address this directly, we added four targeted components that are referenced throughout the responses below:

1. Robust negative controls with 100 uniform random selection replicates for multiple tile budgets, evaluated on identical folds, with confidence intervals and Monte Carlo testing.

2. Quantitative attention concentration and stability metrics for CHIEF, ABMIL, and gated ABMIL, including Lorenz curves, Gini coefficients, and top-k cumulative mass statistics.

3. Attention visualizations comparing CHIEF and ABMIL, with overlays of the top-25 tiles used by EAGLE.

4. A strengthened MIL baseline by adding a gated ABMIL implementation in addition to standard ABMIL under the same feature backbone.

Reviewer #1

Comment: I have read the resubmission carefully, and I still have mixed feelings about the work. Overall, the authors are responsive and have addressed most of my comments on the technical and validation aspects. However, I am still not convinced that this study brings any fundamental innovation to the field. While reducing the computational burden is useful, I am not entirely convinced that this gain is significant, especially given that the reduction stems from a simplistic reduction of the amount of information processed, rather than from algorithmic or principled innovation.

Intuitively, the contextual information in those patches that are excluded by EAGLE should be useful and contribute to the pattern analysis and downstream tasks. For pathologists, the extent of diseased tissue in the context of normal tissue is important. Therefore, I feel that the exclusion of these patches by EAGLE should probably be justified on a more fundamental basis than purely computational speed and performance.

Response: Thank you for this important conceptual critique. We agree that reduced computation alone is not a contribution. The revised manuscript therefore foregrounds a structural claim that we now support quantitatively: under slide-level supervision and realistic cohort sizes, end-to-end attention MIL often fails to learn selective region weighting for subtle biomarker endpoints and behaves close to mean pooling. In this regime, restricting inference to a reproducible, high saliency subset improves statistical conditioning rather than merely reducing workload.

We test this directly using attention concentration metrics. Across tasks and folds, CHIEF induces a stable non-uniform saliency ranking, whereas ABMIL attention remains broadly distributed for most biomarker tasks and becomes meaningfully selective primarily for overt morphology endpoints (for example NSCLC subtyping and CRC sidedness) (**Supplementary Fig. 5**). This conclusion is unchanged after strengthening the baseline with gated ABMIL: gating improves AUROC modestly to 0.723, reaching strong MIL baselines such as STAMP, but remains below EAGLE (0.742) and TITAN (0.740) (**Fig. 3a, ED Fig. 5a**), and does not resolve diffuse attention behavior (**Fig. 4**).

We further exclude the possibility that EAGLE benefits from arbitrary truncation by constructing an empirical null distribution from 100 repeated uniform random tile selections for N = 5, 10, 25, 50, and 100 on identical folds. For every N, CHIEF-based selection exceeds the maximum random replicate, corresponding to Monte Carlo p = 1/101 (**Fig. 3c**). Thus, the gain is not explained by analyzing fewer tiles, but by using a saliency ranking that is consistently more informative than unguided subsampling.

We acknowledge that the contextual extent of disease can be clinically informative in general. Our data indicate that, for the vast majority of tasks in this benchmark, such context is not exploited reliably by end-to-end attention learning under slide-level labels, and therefore including all tiles primarily increases noise exposure rather than improving inference.

Reviewer #4

Comment: This work proposes EAGLE, a lightweight aggregation framework that selects only the top-25 tiles using CHIEF's frozen, task-agnostic attention scores, followed by feature extraction (with Virchow2) and simple averaging. While impressive speed-ups and competitive AUROCs are reported, several fundamental concerns raised by reviewers remain inadequately addressed.

1. The core design (fixed, task-agnostic tile selection) requires strong justification.

The central assumption, that a single set of 25 tiles selected via CHIEF's pretrained attention suffices for all downstream tasks, is highly questionable. In practice, biologically relevant regions vary significantly across tasks: for instance, given the same LUAD WSIs, different tasks like EGFR mutation prediction, TP53 status, and NSCLC often rely on distinct morphological patterns (e.g., tumor-stroma interface vs. necrotic core vs. immune infiltrate). Prior works (e.g., ABMIL) explicitly learn task-specific attention because generic "informative" regions are insufficient.

Notably,

- Reviewer #1 explicitly asked for analysis on how weight distributions among the top-25 tiles behave (e.g., are embeddings dominated by 1–2 tiles? What features drive high attention?). The authors only clarified that aggregation is uniform, without providing the distribution of CHIEF's attention used for selection.

- In the original CHIEF paper (Fig. 2b), its attention is broad and diffuse (high scores span >50% of tissue regions on the provided four cases). Selecting only 25 from this large pool is arbitrary: if CHIEF's attention is truly flat over diagnostically relevant regions (as implied), then any reasonable subset, including uniformly sampled tiles, should perform comparably. Yet the manuscript reports drastically worse performance for random selection (e.g., AUROC drop of ~10%, per Fig. 3c), without reporting confidence interval, despite Reviewer #3 requesting systematic negative controls (100 random replicates). This creates an internal inconsistency: either (i) CHIEF's attention is sharper than disclosed (raising transferability concerns across tasks), or (ii) attention-guided selection does not yield a performance improvement (indicating potentially undertuned baselines, as noted by R2).

Response: We tested the "arbitrary subset" premise with robust negative controls. We repeat uniform random tile selection 100 times for each N = 5, 10, 25, 50, 100. For every N, CHIEF-based selection exceeds the maximum random replicate, yielding Monte Carlo p = 1/101 (**Fig. 3c, Supplementary Table 1**). This excludes the hypothesis that any reasonable subset performs comparably.

We next quantify, rather than assume, the shape of CHIEF attention in our benchmark. CHIEF attention is not flat: the median cumulative mass curves indicate that 8.4% of tiles account for 50% of attention and 20.5% account for 80%, with mean Gini = 0.702 (**Fig. 4e**). To directly address the reviewer's concern about domination by very few tiles, we report top 1 and top 2 mass statistics, which show that the representation is not driven by 1–2 tiles (median top 1 mass = 0.55%, median top 2 mass = 1.1%, **Fig. 4h, Supplementary Table 2**)

Finally, we provide attention heatmaps and explicit overlays of the exact top-25 tiles used by EAGLE. These overlays show that the selected tiles are drawn from the upper tail of a structured attention distribution and cluster in plausible tissue compartments rather than being drawn from broad plateaus (**Fig. 5, Supplementary Fig. 6-7**).

We do not claim that the same 25 tiles are the biologically optimal set for every task. Our claim is narrower and supported by these analyses: under weak supervision, a pretrained saliency prior provides a stable ordering that materially outperforms uniform selection and improves generalization.

Comment: 2. The claimed superiority over Virchow2+ABMIL lacks a fair or mechanistic explanation.

Reviewers #2 and #3 rightly expressed scepticism: "I really would not have expected this method to outperform ABMIL…", "… (EAGLE) essentially an extreme, thresholded implementation of attention-based method…", "the model itself (ABMIL, etc.) should be quite capable of that (filtering out less informative regions). It shouldn't require the patch selection and encoder to do that."

The authors' rebuttal rests on two weak pillars:

(a) Small-data limitation of supervised ABMIL. They claim ABMIL fails due to limited training data. However, there is still no direct evidence on whether task-agnostic large-scale

pretrained attention is better than the task-specific attention in ABMIL. Given the facts that ROIs vary across tasks and the attention module is mainly used for feature aggregation rather than extraction, it is more natural and convincing to use the task-specific attention module.

(b) “Filtering noise improves performance”. However, ABMIL aggregators already down-weight noisy tiles via learned attention. If unweighted averaging of CHIEF-selected tiles beats attention-weighted aggregation over all tiles, it implies CHIEF’s pretrained attention is more discriminative than learned attention, even on out-of-distribution tasks. This contradicts the results from the original CHIEF paper (Fig. 2b) and demands direct visualization (e.g., attention maps across tasks and WSIs) and comparison (e.g., CHIEF’s top-25 tiles regions, CHIEF’s attention map visualization, and task-specific attention map visualization), neither of which is provided.

Response: We agree that this requires mechanistic clarification. We therefore directly compare pretrained CHIEF saliency to task-specific attention learned by ABMIL using the same backbone features and training protocol, and we strengthen the baseline with gated ABMIL.

Quantitatively, ABMIL attention is diffuse for the vast majority of tasks: the median fraction of tiles required to accumulate 50% and 80% attention mass is 44.1% and 75.8%, with mean Gini = 0.087. Gated ABMIL changes these values only modestly (median 50% and 80% mass at 42.0% and 73.6%, mean Gini = 0.104, **Supplementary Table 2, Fig. 4, Supplementary Fig. 5**). In contrast, CHIEF is substantially more concentrated (median 50% and 80% mass at 8.4% and 20.5%, mean Gini = 0.702). This provides direct evidence that, in this benchmark and sample regime, task-specific attention learning frequently collapses toward near uniform aggregation.

Performance results align with this mechanism. Gated ABMIL improves AUROC to 0.723 and reaches strong MIL baselines such as STAMP on average, but remains below EAGLE (**Fig. 3a**, **ED Fig. 5a**). This behavior aligns with recent clinical benchmarking showing that, when using domain specific pathology foundation model embeddings, no single aggregation method is consistently superior across tasks and the inclusion of spatial information yields marginal gains for more challenging endpoints such as outcome prediction or replicative biomarker prediction[1].

We add attention maps comparing CHIEF, ABMIL, and gated ABMIL on the same slides and tasks with explicit top-25 overlays. These show that CHIEF yields a broader dynamic range and clearer spatial concentration, whereas ABMIL variants often distribute weight over large tissue fractions for subtle endpoints (**Fig. 5, Supplementary Fig. 6-7**).

Comment: 3. Interpretability claims may be overstated and not evidence-backed.

The assertion that “using only 25 tiles inherently improves interpretability” conflates sparsity with faithfulness. A sparse selection is only interpretable if the selected tiles are truly diagnostic, yet evaluation remains qualitative (single pathologist without specific metric) and artifact-focused (pen marks are not equivalent to clinical relevance).

Response: We agree that sparsity does not imply faithfulness. We therefore revised the manuscript to remove any claim that using 25 tiles inherently improves interpretability. Our revised claim is narrower and verifiable: EAGLE improves auditability and review efficiency by restricting each prediction to a small, explicitly identified subset of regions whose coordinates can be inspected and reproduced.

Comment: To substantiate EAGLE's contributions, the authors should provide:

1. Robust negative controls: performance of uniform tile selection (25/50/100/…), rather than a single random selection without statistical testing.

2. Attention distribution and stability metrics: Show histograms of CHIEF attention scores and ABMIL attention scores; report % of cases where top-25 tiles dominate for both strategies.

3. Direct visualization and comparison on attention maps: Visualize and compare (i) CHIEF attention heat maps, (ii) ABMIL task-specific attention heat maps, (iii) uniform selection patches location on the heat maps, (vi) CHIEF's top-25 tiles on the heat maps. If these results fail to support the authors' argument, the reliability of EAGLE remains questionable

Response: We appreciate the reviewer's concrete suggestions and have implemented each requested component. Negative controls with 100 random replicates across multiple N are reported with uncertainty and Monte Carlo testing (**Fig. 3c, Supplementary Table 1**). Attention distributions are quantified using Lorenz curves, Gini coefficients, and top-k mass statistics for CHIEF, ABMIL, and gated ABMIL (**Fig. 4, Supplementary Fig. 5, Supplementary Table 2**). Direct visual comparisons include CHIEF and ABMIL attention maps, and explicit CHIEF top-25 overlays (**Fig. 5, Supplementary Fig. 6-7**).

1. *Chen, S. et al. Benchmarking embedding aggregation methods in computational pathology: a clinical data perspective. Proc. Mach. Learn. Res. 254, 38–50 (2024).*

Reviewer #1

Comment: The manuscript and the presentation of results have been improved; however, I feel that the fundamental methodology remains largely unchanged.

I am a proponent of sparse decision-making, but sparsity is usually a consequence of limited measurement data. When data are abundant or oversampled, researchers typically sparsify based on solid theoretical frameworks. The approach taken in this work appears purely empirical, lacking a well-established theoretical foundation.

While EAGLE’s strategy of analyzing only 25 selected tiles significantly accelerates the workflow, it introduces a latent risk of missing critical clinical information. In complex malignancies, sparse sampling—representing only a fraction of the total tissue—may fail to capture the full diagnostic landscape. This contrasts with standard clinical practice, where pathologists must examine the entire Whole Slide Image (WSI), and often multiple levels or blocks, to ensure a comprehensive diagnosis. Indeed, clinical pathology relies on a holistic evaluation of the tumor microenvironment, including stromal interactions and vascular invasion.

EAGLE’s "selective focus" mimics the human eye but lacks the global spatial context provided by dense-sampling models. In cases where diagnostic clues are subtle or scattered, a sparse-sampling approach might overlook information that a full-scale review would catch. Furthermore, despite its superior average performance in tasks requiring minimal morphological information, EAGLE is outperformed by models like TITAN in specific categories—particularly morphology-heavy tasks such as lung cancer subtyping. This suggests that for tasks requiring the integration of macro-scale structural features and long-range spatial dependencies, heavy-weight slide encoders that process the full image still maintain a competitive edge.

Response: Thank you for this thoughtful comment. We agree that the efficiency gains of sparse region selection must be interpreted together with its potential limitations. In the revised Discussion, we now state more explicitly that the advantages of EAGLE are task-dependent and that dense slide encoders may remain competitive or superior for morphology-heavy endpoints requiring broader architectural context or longer-range spatial dependencies, as illustrated by TITAN in non-small cell lung cancer subtyping. We also clarify that any sparse-sampling strategy carries a residual risk of missing rare, spatially dispersed, or globally contextual cues that a dense whole-slide encoder or full human review could capture. At the same time, we emphasize that the strong performance of EAGLE in our benchmark reflects a favorable operating point for many clinically relevant tasks rather than a claim that sparse sampling is universally optimal. We have revised the Discussion accordingly.

Reviewer #4

Comment: The authors provide a concrete analysis of EAGLE's underlying mechanisms. In particular, the analyses of attention concentration and the attention-map visualizations for ABMIL and EAGLE largely address my concerns regarding the tile-selection strategy.

One minor point still warrants discussion. While the manuscript does not claim that selecting the same 25 tiles is biologically optimal for every task, the results in Supplementary Fig. 3a suggest that this choice performs well across different task types. Although I understand that involving additional pathologists to assess the biological relevance of the selected tiles may be challenging, a discussion of this somewhat counterintuitive observation would be helpful.

Response: Thank you for this helpful suggestion. We agree that the strong performance of a fixed 25-tile budget across diverse endpoints warrants explicit discussion. In the revised Discussion, we now clarify that this result should be interpreted as a robust empirical operating point in our benchmark rather than as a biologically universal optimum. We further discuss a plausible explanation for this observation, namely that histopathology contains substantial spatial redundancy and that CHIEF ranking appears to recover much of the predictive signal early, while also noting that the optimal tile budget is likely to remain task-dependent. We have revised the Discussion accordingly.
